# Integrative metabolomic and transcriptomic analyses reveal the regulatory mechanisms underlying the biosynthesis of flavonoid and terpenoid metabolites in different tissues of *Canavalia gladiata*

**DOI:** 10.3389/fpls.2026.1792177

**Published:** 2026-04-15

**Authors:** Yue Gao, Yucheng Yang, Yumeng Zhang, Yi Zhao, Zixuan Fan

**Affiliations:** College of Food Science and Technology, Wuhan Business University, Wuhan, China

**Keywords:** *Canavalia gladiata*, flavonoids, metabolome, terpenoids, transcriptome

## Abstract

**Introduction:**

*Canavalia gladiata* (*C. gladiata*) is an important medicinal and edible plant. However, systematic research on the distribution of metabolites in different tissues of *C. gladiata* and their potential transcriptional regulatory mechanisms remain poorly understood.

**Methods:**

We performed integrated metabolomic and transcriptomic analyses across five tissues (roots, stems, leaves, seeds, and fruit pericarps) of *C. gladiata*, combined with antioxidant capacity and bioactive component content assays, to dissect the regulatory networks of flavonoid and terpenoid biosynthesis.

**Results:**

Seeds exhibited the highest antioxidant activity and total phenolic content, whereas leaves accumulated the highest levels of total flavonoids and terpenic acids. A total of 4,405 DAMs and 25,597 DEGs were identified, revealing pronounced tissue-specific metabolic and transcriptional divergence. Flavonoid and terpenoid biosynthesis pathways were significantly enriched in comparisons between seeds and other tissues. Key structural genes, including *4CL, CHS, FLS, HMGR*, and *DXS*, displayed strong tissue-specific expression patterns. Co-expression network analysis identified candidate regulatory modules, highlighting *MYB*, *bHLH*, and *MYC2* transcription factors as central regulators of flavonoid and terpenoid metabolism in seeds and fruit pericarps.

**Discussion:**

This study provides the first comprehensive landscape of tissue-specific flavonoid and terpenoid metabolism in *C. gladiata*, offering a theoretical foundation and valuable genetic resources for the targeted exploitation of its bioactive components and molecular breeding.

## 1 Introduction

*Canavalia gladiata* (sword bean) is an underutilized legume with both edible and medicinal value, cultivated widely across Asia and other tropical regions (Ekanayake et al., 2000).The plant is rich in bioactive compounds, including flavonoids, terpenoids, and phenolic acids, which contribute to its diverse pharmacological properties, such as antioxidant, anti-inflammatory, and antitumor effects (Laija et al., 2010; Li et al., 2011; Rokkam et al., 2023; Qian et al., 2025). Notably, the total flavonoid content of *C. gladiata* is significantly higher than that of soybean, highlighting its strong potential as a high-value functional food source (Kim et al., 2012; Gan et al., 2017). Pharmacological studies have further demonstrated that different tissues of *C. gladiata* exhibit distinct pharmacological activities and clinical application potentials (Gan et al., 2016; Pal et al., 2022; Qian et al., 2025; Hwang et al., 2025). For instance, a comparative metabolomic analysis identified 31 metabolites in the leaves and seeds, highlighting both tissues as significant repositories of antioxidant components (More et al., 2022). Extracts from sword bean pods have also been shown to promote osteoblast differentiation via activation of the *BMP2/SMAD/RUNX2* signaling pathway, supporting its beneficial role in bone health (Hwang et al., 2023). Nevertheless, systematic research on the tissue-specific enrichment of functional metabolites, along with their underlying biosynthetic and regulatory mechanisms, remains severely limited in *C. gladiata*.

Flavonoids and terpenoids represent two predominant classes of plant secondary metabolites, playing indispensable roles in defense responses and environmental adaptation (Li et al., 2023; Wu et al., 2023; Câmara et al., 2024).Flavonoids are synthesized via the phenylpropanoid pathway, while terpenoids originate from the mevalonate (MVA) or methylerythritol phosphate (MEP) pathways (Movahedi et al., 2022; Zhuang et al., 2023).Despite their distinct biochemical origins, biosynthesis of both metabolite classes is subject to sophisticated, tightly coordinated transcriptional regulation. R2R3-MYB transcription factors can independently activate early flavonoid biosynthetic genes, such as *CHS*, *CHI*, *F3H* and *FLS*, thereby controlling the initial flux through the phenylpropanoid and flavonoid pathways (Cao et al., 2024). In contrast, coordinated activation of late flavonoid biosynthetic genes, such as *DFR* and *ANS*, typically requires the formation of *MYB–bHLH–WD40 (MBW)* transcriptional complexes (Bulanov et al., 2025). Similarly, terpenoid accumulation is modulated by a diverse set of TFs including *MYC2*, *WRKY*, and *AP2/ERF* family members, which directly target and regulate key enzyme-encoding genes in the MVA and MEP pathways (Singh et al., 2024). Importantly, accumulating evidence from medicinal plant studies indicates that the regulatory mechanisms governing flavonoid and terpenoid biosynthesis exhibit pronounced tissue specificity (Zhang et al., 2022; Ma et al., 2024; Dong et al., 2025; Wang et al., 2025; Xu et al., 2025). Distinct transcription factor repertoires and co-expression modules have been identified in roots, leaves, stems, and flowers, resulting in organ-specific activation of flavonoid and terpenoid biosynthetic pathways (Zhang et al., 2022; Dong et al., 2025; Wang et al., 2025). Consequently, the tissue-dependent assembly of transcriptional complexes and spatial expression of structural genes collectively shape differential metabolite accumulation patterns.

While the biosynthetic and regulatory frameworks of flavonoids and terpenoids are well-characterized in many model and medicinal plants, the corresponding regulatory architecture in *C. gladiata* remains largely unexplored. Integrated transcriptomic and metabolomic analyses offer a systematic approach to identifying key structural genes and regulators, thereby elucidating complex metabolic networks (Li et al., 2020a; Shen et al., 2023; Fan et al., 2024). In this study, we conducted the first comprehensive investigation of the metabolomic and transcriptomic profiles across five distinct tissues (roots, stems, leaves, seeds, and fruit pericarps) of *C. gladiata*. Our objectives were to: (1) characterize tissue-specific metabolite accumulation patterns, focusing on flavonoids and terpenoids; (2) identify key differentially expressed genes (DEGs) and transcription factors (TFs) associated with these pathways; and (3) construct integrated regulatory networks linking TFs to structural genes and metabolites. This work provides a theoretical foundation for understanding the molecular basis of metabolite synthesis in *C. gladiata* and offers valuable genetic resources for its functional optimization and molecular breeding.

## 2 Materials and methods

### 2.1 Plant materials and tissue collection

Plant materials of *C. gladiata* were collected from Shaoshan, Hunan, China (112°31′17.9″E, 27°55′6.0″N; elevation=126 m) during the fruit ripening stage in October 2024. The collected samples were categorized into five groups based on tissue type: roots (R), stems (St), leaves (L), seeds (Se), and fruit pericarps (FP). The flowering period of *C. gladiata* typically spans two months. Flowers were tagged at 0 days post-anthesis (0 DPA), and seeds and fruit pericarps were harvested at 25 DPA. All samples, selected for being free of decay, mold, and mechanical damage, were immediately snap-frozen in liquid nitrogen and stored at −80 °C. Four biological replicates were used for metabolomic extraction, while three biological replicates were employed for transcriptome analysis and the determination of physicochemical indicators.

### 2.2 Determination of antioxidant capacity and physicochemical indicators

The antioxidant capacity of *C. gladiata* extracts was evaluated by three commonly used methods, and each assay was performed using the corresponding commercial kit following the manufacturer’s protocols. Specifically, the 2,2-diphenyl-1-picrylhydrazyl (DPPH) radical scavenging assay was measured using the DPPH assay kit (Macklin, T931095); the 2,2’-azino-bis (3-ethylbenzothiazoline-6-sulfonic acid) (ABTS) radical cation decolorization assay was conducted with the ABTS assay kit (Macklin, T931096); and the ferric reducing antioxidant power (FRAP) assay was determined using the FRAP assay kit (Macklin, T931094).

Total phenolic content (TPC) (Solarbio, BC1330) and total flavonoid content (TFC) (Macklin, T930799) were determined according to the manufacturers’ instructions. The total terpenic acid content was measured in accordance with the Agricultural Industry Standard of the People’s Republic of China (NY/T 3676-2020) (Ministry of Agriculture and Rural Affairs of the People’s Republic of China, 2020).

### 2.3 Metabolome analysis

The samples were gradually thawed at 4 °C, and an appropriate aliquot (50–100 mg) of each sample was precisely weighed and transferred into a centrifuge tube. Subsequently, 1 mL of a pre-cooled extraction solvent (a mixture of water, acetonitrile, and isopropyl alcohol in a ratio of 1:1:1, v/v/v) was added. The mixture was homogenized for 60 seconds and subjected to low-temperature ultrasonic extraction for 30 minutes. Following extraction, the samples were centrifuged at 12,000 rpm for 10 minutes at 4 °C. To facilitate protein precipitation, the samples were allowed to stand at -20 °C for 1 hour and then centrifuged again under the same conditions. The resulting supernatant was vacuum-dried, reconstituted in 0.2 mL of 30% acetonitrile solution, and homogenized. Finally, the solution was centrifuged at 14,000 rpm for 15 minutes at 4 °C, and the supernatant was collected for subsequent computer-based analysis.

A Vanquish UPLC system (Thermo Fisher Scientific, USA) was used in conjunction a Q Exactive HFX mass spectrometer (Thermo Fisher Scientific, USA).The samples were injected onto a Waters HSS T3 column (100 × 2.1 mm, 1.8μm) maintained at 40°C, with a flow rate of 0.3 mL/min and an injection volume of 2μL.The mobile phase consisted of Milli-Q water containing 0.1% formic acid (eluent A) and acetonitrile containing 0.1% formic acid (eluent B). The gradient elution program was as follows: 0 min phase A/phase B (100:0, v/v), 1 min phase A/phase B (100:0, v/v), 4 min phase A/phase B (40:60, v/v), 6.5 min phase A/phase B (5:95, v/v), 6.6 min phase A/phase B (100:0, v/v), 8.0 min phase A/phase B (100:0, v/v). A positive/negative polarity Q ExactiveTM HF-X mass spectrometers were set as follows: spray voltage +3 kV/-2.8 kV; capillary temperature of 350 °C, sheath gas flow rate of 40 arb, and aux gas flow rate of 10 arb was used. The temperature of the ion transport tube was set at 320°C. The scanning range for the primary mass spectrometry was set from 70 to 1050 Da, with a primary resolution of 70,000 and a secondary resolution of 17,500.

Raw MS data were acquired using Xcalibur 4.1 software (Thermo Fisher Scientific, USA) and processed with Progenesis QI (Waters Corporation, USA) for feature extraction and alignment. Principal Component Analysis (PCA) was performed using SIMCA-P 14.1 (Umetrics, Sweden). Pearson’s Correlation Coefficients (PCC) between samples were calculated with the “cor” function in the R (version 3.5.1) and the results were visualized as heatmaps. Differentially accumulated metabolites (DAMs) were identified based on a fold change (FC) ≥ 2 or ≤ 0.5 and a variable importance in projection (VIP) ≥ 1. Metabolites were annotated using the KEGG Compound database (http://www.kegg.jp/kegg/compound/), followed by mapping of the annotated metabolites to the KEGG Pathway database (http://www.kegg.jp/kegg/pathway.html).

### 2.4 RNA extraction and transcriptome analysis

Total RNA was extracted from the plant materials using pre-frozen TRIzol reagent (Thermo Scientific, 15596026CN) in accordance with the manufacturer’s instructions. The quantity and quality of total RNA was extracted and assessed using NanoDrop 2000 (Thermo Fisher Scientific, USA). The mRNA is then enriched using Oligo dT magnetic beads and fragmented into small pieces of approximately 300 bp. Double-stranded cDNA is synthesized through reverse transcription and ligated with Y-shaped adapters. Finally, after fragment selection, PCR amplification, and library purification, high-throughput sequencing of mRNA is performed using the DNBSEQ-T7 sequencer (MGI Tech, China). The clean reads were then mapped to the reference genome (GCA_037954105.1) using the STAR software (Dobin and Gingeras 2015). Differential expression analysis of gene expression was performed using DESeq2 (Love et al., 2014) with a threshold of |log2FoldChange|> 1 for DEGs and a significance P-value < 0.05. Genes were aligned with protein sequences in the NR database and SwissProt database via BLAST (evalue ≤ 1e-5), and the alignment results were used as annotations for the corresponding genes. GO and KEGG enrichment analyses of DEGs were performed using the clusterProfiler R package (Xu et al., 2024). Transcription factors (TFs) were predicted from the assembled transcriptome of *C. gladiata* via the Plant Transcription Factor Database (PlantTFDB, http://planttfdb.gao-lab.org/).

### 2.5 Integrated analysis of transcriptome and metabolome

The screened DAMs and DEGs were mapped to the KEGG pathway database (www.kegg.jp/kegg/kegg.html), thereby identifying flavonoid- and terpenoid-related KEGG pathways at the transcriptome and metabolome levels. To construct a metabolite-gene expression regulatory network, we first screened genes significantly correlated with each DAMs according to the criteria of absolute Pearson correlation coefficient > 0.8 and p-value < 0.05. Subsequently, K-means analysis was performed on the transcriptome and metabolome data of different tissue samples using R software and its stats package (version 4.2.0). Finally, a regulatory network involving transcription factors, structure genes and metabolites was constructed via the Pearson correlation algorithm.

### 2.6 Quantitative real-time PCR

To validate the RNA-seq data, ten genes were selected for qRT-PCR. Gene-specific primers were designed using Primer Premier 5 software. cDNA was synthesized from 2 μg of total RNA using a cDNA synthesis kit (Thermo Scientific, M1682). qRT-PCR was performed in technical triplicates using PowerUp SYBR Green Master Mix (Yeasen, 11202ES03) on a QuantStudio 6 Flex Real-Time PCR system (Applied Biosystems, USA). Relative expression levels were calculated using the 2^-ΔΔCt^ method, with actin11 (gene-VNO77_21608) serving as the internal reference gene.

### 2.7 Statistical analysis

One-way analysis of variance (ANOVA) was performed using IBM SPSS Statistics (Version 25.0) to determine statistical significance. For all analyses, the significance threshold was set at *p* < 0.05.

## 3 Results

### 3.1 Antioxidant capacity and physicochemical properties of different tissues in *C. gladiata*

**Figure 1:**
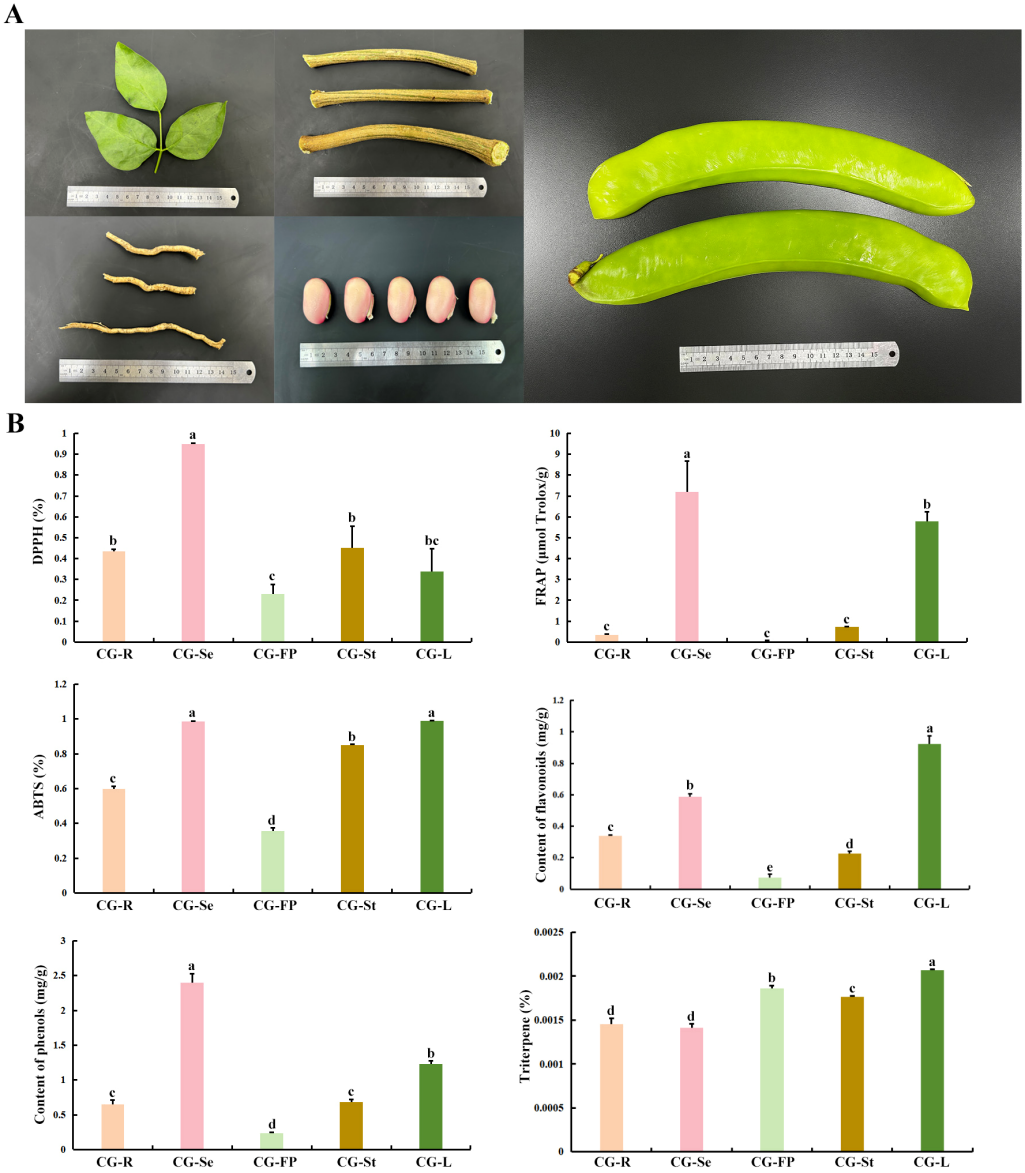
Physiological and biochemical indices of different tissues of *C. gladiata*. **(A)** Collected samples of roots, stems, leaves, seeds and fruit pericarps of *C. gladiata*; **(B)** Antioxidant activity indices (DPPH, FRAP and ABTS), total flavonoid content, total terpenic acid content, and total phenolic acid content in different tissues. Statistical significance was determined by ANOVA and Duncan’s test *post hoc* analysis. Different lowercase letters indicate significant differences between groups (*P* < 0.05).

The medicinal value of *C. gladiata* varies across different tissues due to distinct profiles of bioactive components (Gan et al., 2016; Pal et al., 2022). To evaluate these differences, we determined antioxidant indices (DPPH, FRAP, and ABTS) in five tissues (roots, stems, leaves, seeds, and fruit pericarps). The results manifested that seeds exhibited the highest antioxidant activity, followed by leaves, while the fruit pericarps showed the lowest (**Figures 1A, B**). Consistent with these findings, seeds and leaves contained the highest concentrations of total flavonoids and total phenols. Furthermore, total terpenic acids were relatively enriched in leaves and fruit pericarps, but remained low in roots and seeds (**Figure 1B**).

### 3.2 Metabolomic analysis of different tissues in *C. gladiata*

**Figure 2:**
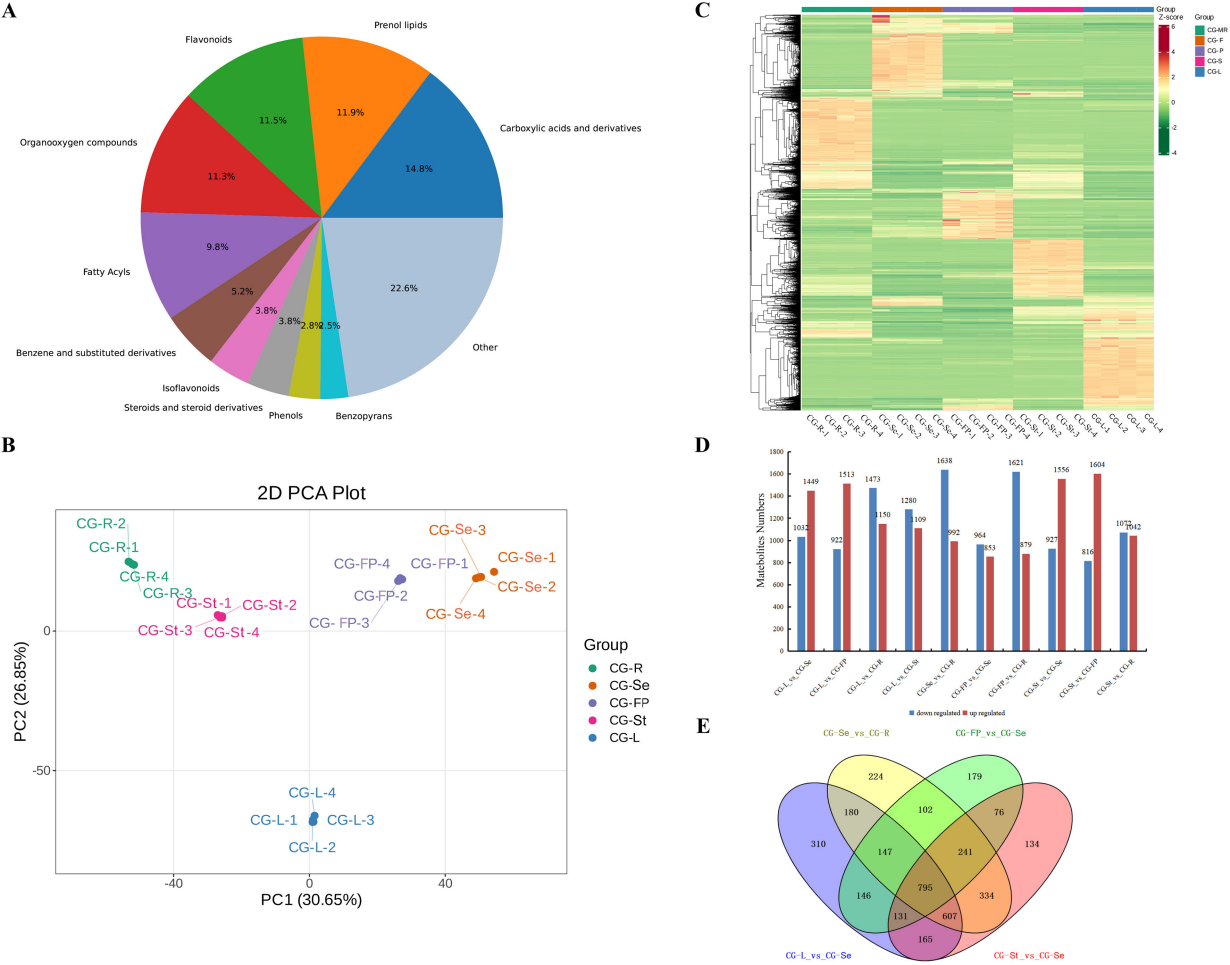
Metabolome analysis of different tissues of *C. gladiata*. **(A)** Pie chart of classification statistics for all metabolites; **(B)** PCA analysis of metabolome data from different tissues, where each point represents a sample and samples in the same group are marked with the same color; **(C)** Heatmap of hierarchical clustering analysis based on differential metabolites in different tissues, with red indicating high metabolite content and green indicating low metabolite content in samples; **(D)** Number of differential accumulated metabolites in pairwise comparisons between different groups, with red representing up-regulated metabolites and blue representing down-regulated metabolites; **(E)** Venn diagram showing the distribution of differential metabolites in different tissues of *C. gladiata*, where each circle represents a comparison group. The overlapping numbers between circles indicate the number of common metabolites among comparison groups, while the numbers in non-overlapping parts of circles indicate the number of unique metabolites in each comparison group.

To further investigate the spatiotemporal distribution of bioactive components, metabolomic profiling of diverse tissue samples was performed using liquid chromatography-mass spectrometry (LC-MS), and a total of 4,645 metabolites were identified (**Supplementary Table 1**). Among these metabolites, the number of metabolites annotated by the KEGG database was 1,062, accounting for approximately 22.9% of the total identified metabolites (**Supplementary Table 1**). Based on their skeleton structures, these metabolites were categorized into 11 distinct classes. The most abundant class of detected metabolites was Carboxylic acids and derivatives (accounting for 14.8%), followed by Prenol lipids (11.9%), Flavonoids (11.5%) and Organooxygen compounds (11.3%) (**Figure 2A**). Subsequently, unsupervised principal component analysis (PCA) was conducted for multivariate statistical analysis to identify the overall metabolic differences among different tissue samples of *C. gladiata* (**Figure 2B**). The results showed that principal component 1 (PC1) and principal component 2 (PC2) accounted for 30.65% and 26.85% of the total variance contribution rate, respectively, which could effectively distinguish samples from different tissues. The replicate samples of each tissue exhibited low variability, whereas a distinct differentiation pattern was observed between different tissue types. The metabolite accumulation patterns showed the highest similarity between roots and stems, as well as between seeds and fruit pericarps, while leaves displayed the most significant differences compared with other tissue.

### 3.3 Analysis of differentially accumulated metabolites

Based on the criteria of fold change (FC) ≥ 2 or ≤ 0.5 and variable importance in projection (VIP) ≥ 1, a total of 4,405 significantly DAMs were identified from pairwise comparisons between groups (**Supplementary Table 2**). The largest number of DAMs was detected in the comparison between seeds and roots, while the fewest were identified between seeds and fruit pericarps (**Figure 2D**). Venn diagram analysis revealed 795 DAMs shared across the four comparisons involving seeds, suggesting these metabolites may play unique roles in seed development (**Figure 2E**). Cluster heatmap analysis further confirmed tissue-specific spatial distribution patterns (**Figure 2C**).

The top 20 DEMs were identified in each comparison group (**Supplementary Figure 3**). In the comparison between seeds and roots, 6-Ketoestriol, Cussovantoside B and Myricetin 3-Robinobioside were significantly up-regulated, while the flavonoid metabolites 5, 7-Dihydroxy-3, 6-dimethoxyflavone, 5,6-Dihydroxy-4’-Methoxyflavanone and 3,7-Dihydroxy-3’,4’-Dimethoxyflavone were significantly down-regulated. In the comparison between leaves and seeds, the polyhydroxy unsaturated fatty acids (5r,6z,8e,10e,12s,14z)-5,12,20,20-Tetrahydroxyicosa-6,8,10,14-Tetraenoic Acid and (5r,6z,8e,12r,14z)-5,12,20,20,20-Pentahydroxyicosa-6,8,14-Trienoic Acid were significantly down-regulated, whereas the flavonoid metabolite Phloretin 3’,5’-Di-C-Glucoside was significantly up-regulated. In the comparisons between stems and seeds, as well as between fruit pericarps and seeds, the flavonoid metabolites Sophoronol B and 5,6-Dihydroxy-4’-Methoxyflavanone were both significantly up-regulated.

For the DAMs identified in each comparison group, KEGG enrichment analysis was performed to elucidate their associated metabolic pathways (**Supplementary Figure 4**). The results showed that the Flavone and flavonol biosynthesis and Isoflavonoid biosynthesis pathways were significantly enriched in the comparisons between stems and seeds, as well as between seeds and roots (P < 0.05). In addition, the Flavone and flavonol biosynthesis and Diterpenoid biosynthesis pathways were significantly enriched in the comparison between fruit pericarps and seeds (P < 0.05). These results suggested that the flavonoid and terpenoid biosynthesis pathways might be the key pathways responsible for the metabolic differences between seeds and other tissues.

### 3.4 Transcriptomic analysis of different tissues in *C. gladiata*

**Figure 3:**
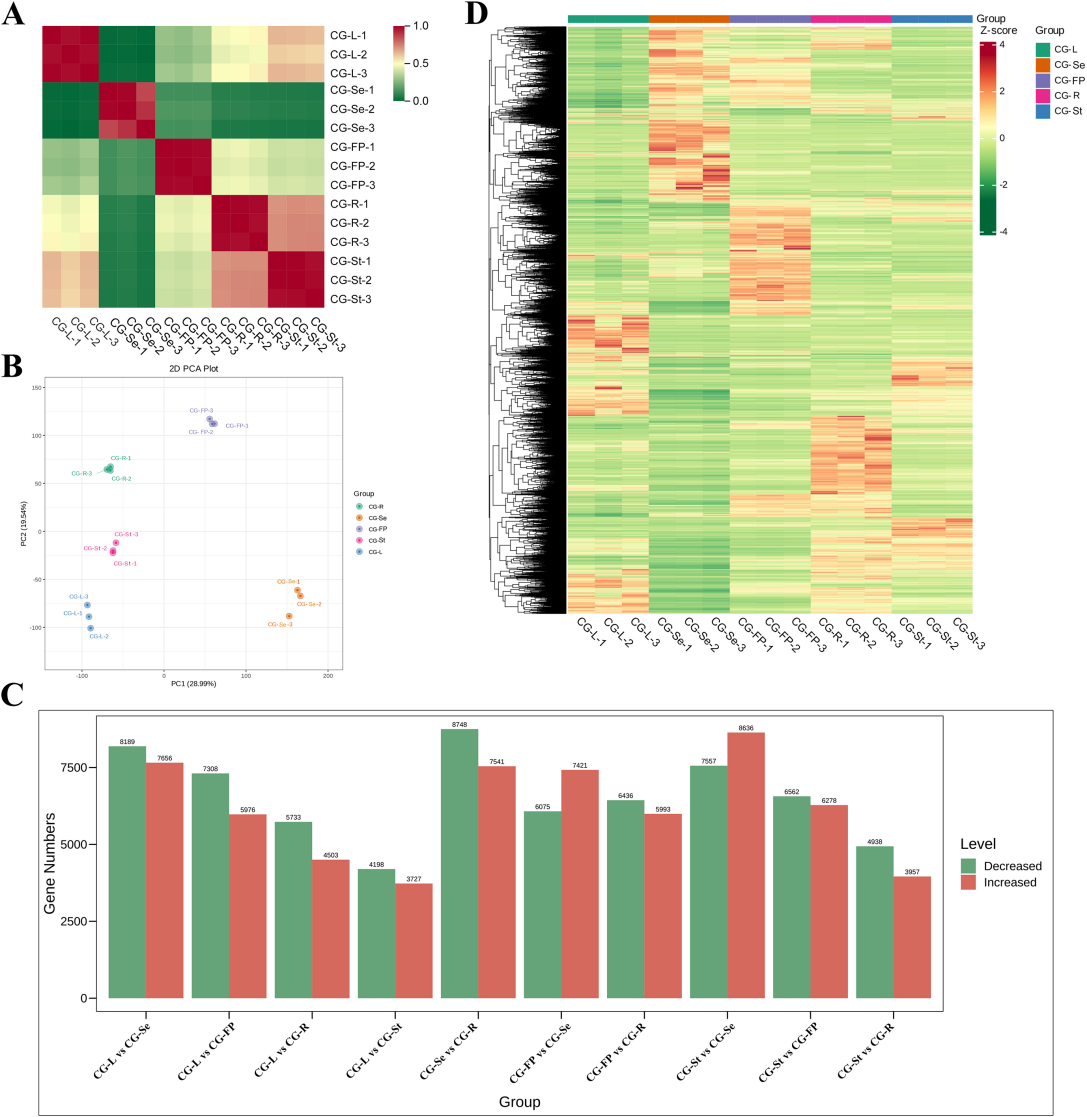
Transcriptome analysis of different tissues of *C. gladiata*. **(A)** Correlation analysis of *Canavalia gladiata* Samples; **(B)** PCA analysis of transcriptome data from different tissues, where each point represents a sample and samples in the same group are marked with the same color; **(C)** Statistical chart of the number of differential genes; **(D)** Heatmap of hierarchical clustering analysis based on DEGs in different tissues, with red indicating high gene expression and green indicating low gene expression in samples.

To explore the molecular mechanisms underlying the differences in antioxidant activity and metabolome among different tissues of *C. gladiata*, transcriptomic analysis was performed. A total of five RNA sequencing libraries were constructed from samples of different tissues (with three biological replicates per sample). After removing low-quality reads, we obtained a total of 133.4 Gb of clean data, with an average of 8.89 Gb per sample (**Supplementary Table 3**). The clean reads of each sample were mapped to the reference genome, and the mapping rates ranged from 84.6% to 96.4% (**Supplementary Table 4**). The Q30 value exceeded 96.33%, and the GC content was 41% (**Supplementary Table 3**), indicating that the RNA sequencing results were of extremely high quality. A total of 49,074 genes were detected to be expressed in the samples (**Supplementary Table 5**). The overall gene expression profiles of different samples are shown in **Supplementary Figure 5**. The Pearson correlation coefficients among the biological replicates of the five tissue samples were relatively high (0.9–1), suggesting that the transcriptomic data had good reproducibility (**Figure 3A**). The principal component analysis plot showed clear separation among different tissue types, and the biological replicates within each sample clustered closely together. The gene expression patterns in leaves, stems and roots exhibited certain similarities, whereas the gene expression pattern in seeds was significantly different from that in other tissues (**Figure 3B**).

### 3.5 Identification of differentially expressed genes

Based on the criteria of |log_2_(fold change)| ≥ 1 and false discovery rate (FDR) ≤ 0.05, a total of 25,597 DEGs were identified (**Supplementary Table 6**). The largest number of DEGs was observed in the comparison between seeds and roots, with 16,289 genes identified in total, among which 7,541 genes were up-regulated and 8,748 genes were down-regulated. This was followed by the comparisons between seeds and stems, as well as between seeds and leaves, where 16,193 and 15,845 DEGs were identified, respectively. On the other hand, the smallest number of DEGs was detected between stems and leaves, with only 7,925 DEGs, including 3,727 up-regulated genes and 4,198 down-regulated genes (**Figure 3C**). To further investigate the expression patterns of DEGs across different tissues, hierarchical clustering analysis was performed. The clustering results showed that the gene expression patterns among biological replicates within each sample group were highly similar, while some DEGs exhibited tissue-specific expression patterns (**Figure 3D**).

As core components of the plant gene expression regulatory network, transcription factors play crucial roles in mediating plant growth and development processes as well as regulating secondary metabolite biosynthesis. Transcription factor annotation of the DEGs identified a total of 1,330 transcription factors, which were classified into 53 families. Among these families, the bHLH family was the most abundant (accounting for 11.2%), followed by the ERF (8.4%), NAC (6.2%), WRKY (5.8%), C2H2 (5.3%) and MYB (5.0%) families (**Supplementary Table 6**; **Supplementary Figure 6**).

### 3.6 GO and KEGG enrichment analyses of differentially expressed genes

**Figure 4:**
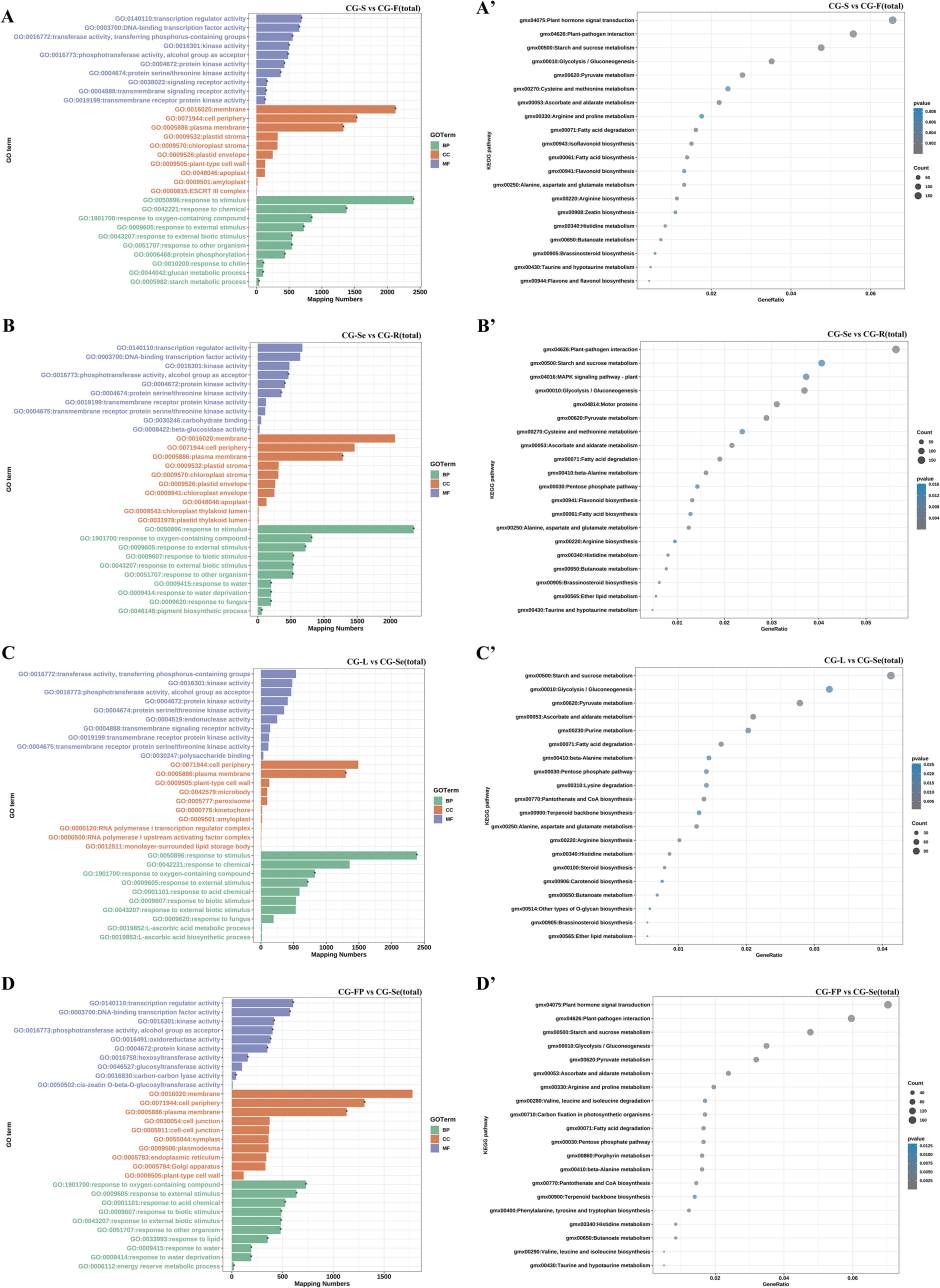
GO and KEGG enrichment analyses of differentially expressed genes (DEGs). **(A)** GO enrichment analysis of DEGs between stems and seeds; A’. KEGG enrichment pathway analysis of DEGs between stems and seeds; **(B)** GO enrichment analysis of DEGs between seeds and roots; B’. KEGG enrichment pathway analysis of DEGs between seeds and roots; **(C)** GO enrichment analysis of DEGs between leaves and seeds; C’. KEGG enrichment pathway analysis of DEGs between leaves and seeds; **(D)** GO enrichment analysis of DEGs between fruit pericarps and seeds; D’. KEGG enrichment pathway analysis of DEGs between fruit pericarps and seeds.

To analyze the functions of the DEGs, GO enrichment analysis was performed. GO enrichment analysis categorizes gene functions into three categories: biological process, cellular component, and molecular function. The results showed that significant differences were observed between seeds and roots, stems, leaves in terms of transcription regulator activity (GO:0140110), DNA-binding transcription factor activity (GO:0003700), kinase activity (GO:0016301), membrane (GO:0016020), cell periphery (GO:0071944), plasma membrane (GO:0005886), response to stimulus (GO:0050896), response to chemical (GO:0042221) and other functional terms (**Figure 4**).

To explore the gene functional pathways associated with the DEGs across different tissues, we conducted KEGG Enrichment Analyses. We selected the top 20 most important pathways and visualized them using scatter plots (**Figure 4**). The results revealed that DEGs were significantly enriched in metabolic pathways including Starch and sucrose metabolism (gmx00500), Glycolysis/Gluconeogenesis (gmx00010), Pyruvate metabolism (gmx00620), Fatty acid degradation (gmx00071), and Alanine, aspartate and glutamate metabolism (gmx00250). In the comparisons between seeds and other tissues, DEGs were enriched in pathways such as Flavonoid biosynthesis (gmx00941), Isoflavonoid biosynthesis (gmx00943), Flavone and flavonol biosynthesis (gmx00944) and Terpenoid backbone biosynthesis (gmx00900), indicating the characteristics of tissue-specific differentiation of secondary metabolites in *C. gladiata*.

### 3.7 Correlation analysis of transcriptome and metabolome expression data

Flavonoids and terpenoids are important secondary metabolites in *C. gladiata*, and they possess significant value in antioxidation, antidiabetic effects and anti-inflammatory activity (Pal et al., 2022). To dissect the molecular regulatory network of flavonoids and terpenoids in *C. gladiata*, we first performed a correlation analysis between the 348 screened DAMs belonging to flavonoids, isoflavonoids and terpenoids and the 25,597 DEGs. Genes significantly correlated with each DAMs were screened according to the criteria of absolute Pearson correlation coefficient > 0.8 and p-value < 0.05. A total of 22,615 genes co-regulated with at least one metabolite were identified (**Supplementary Table 7**).

**Figure 5:**
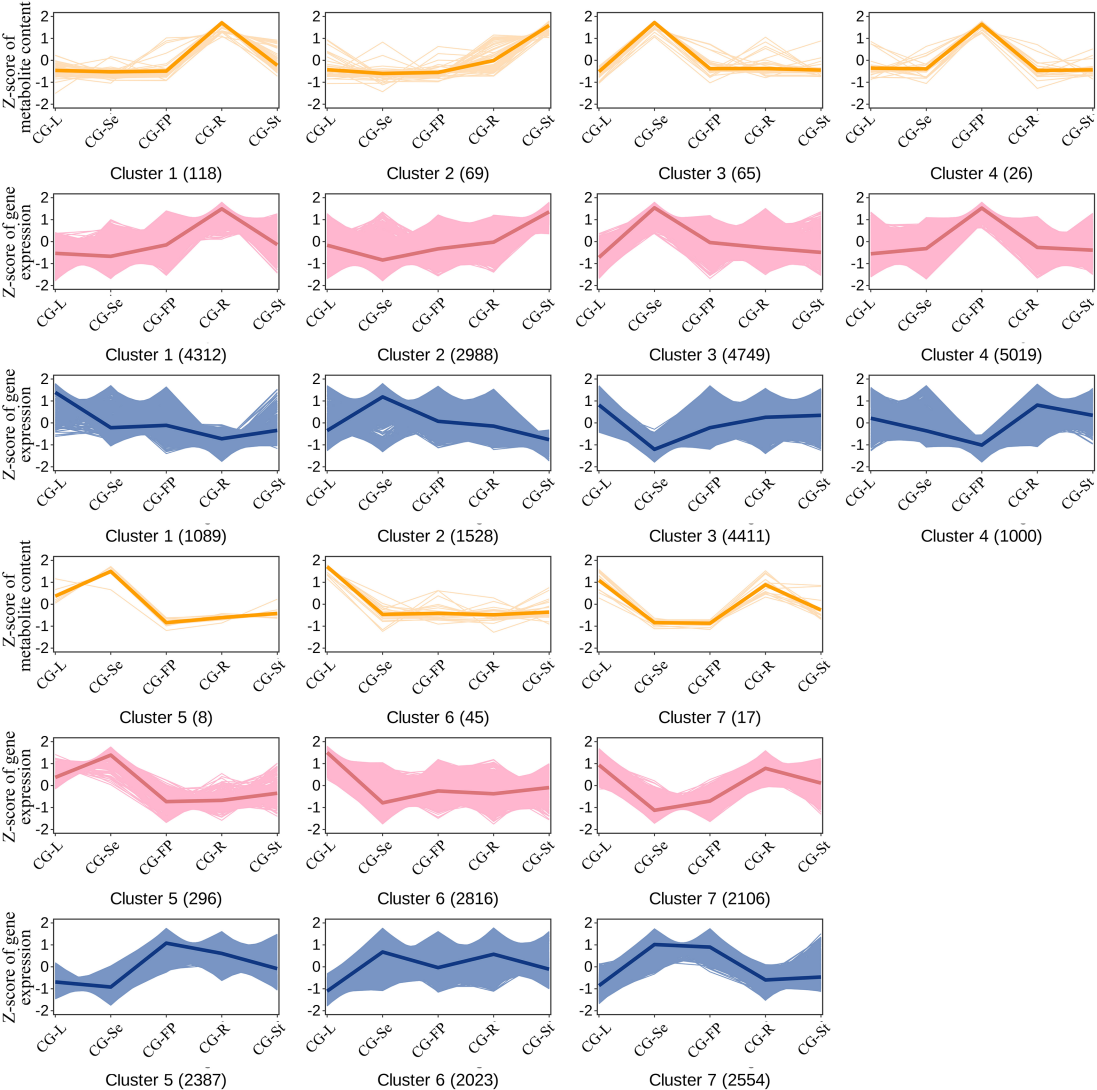
K-means clustering plot of differentially expressed genes and differentially accumulated metabolites. The abscissa represents sample names. The ordinate represents the Z-score normalized values of metabolites (red) and genes (blue). The number in each parenthesis represent the quantity of metabolites and genes in the corresponding cluster.

Furthermore, the 348 metabolites and 22,615 genes were divided into 7 co-expression clusters via k-means analysis, which exhibited consistent or opposite expression patterns across different tissues of *C. gladiata* (**Figure 5**; **Supplementary Table 7**). The significantly correlated metabolites and genes within the same co-expression cluster indicated a closer expression regulatory relationship between them. Through the analysis of these 7 clusters, we identified tissue-enriched clusters: for instance, Cluster 1 was mainly enriched in roots, Cluster 2 in stems, Cluster 3 and Cluster 5 in seeds, Cluster 4 in fruit pericarps, and Cluster 6 in leaves. In addition, several flavonoid and terpenoid metabolites in Cluster 7 were simultaneously enriched in both leaves and roots.

### 3.8 Analysis of the regulatory network of flavonoid metabolites

**Figure 6:**
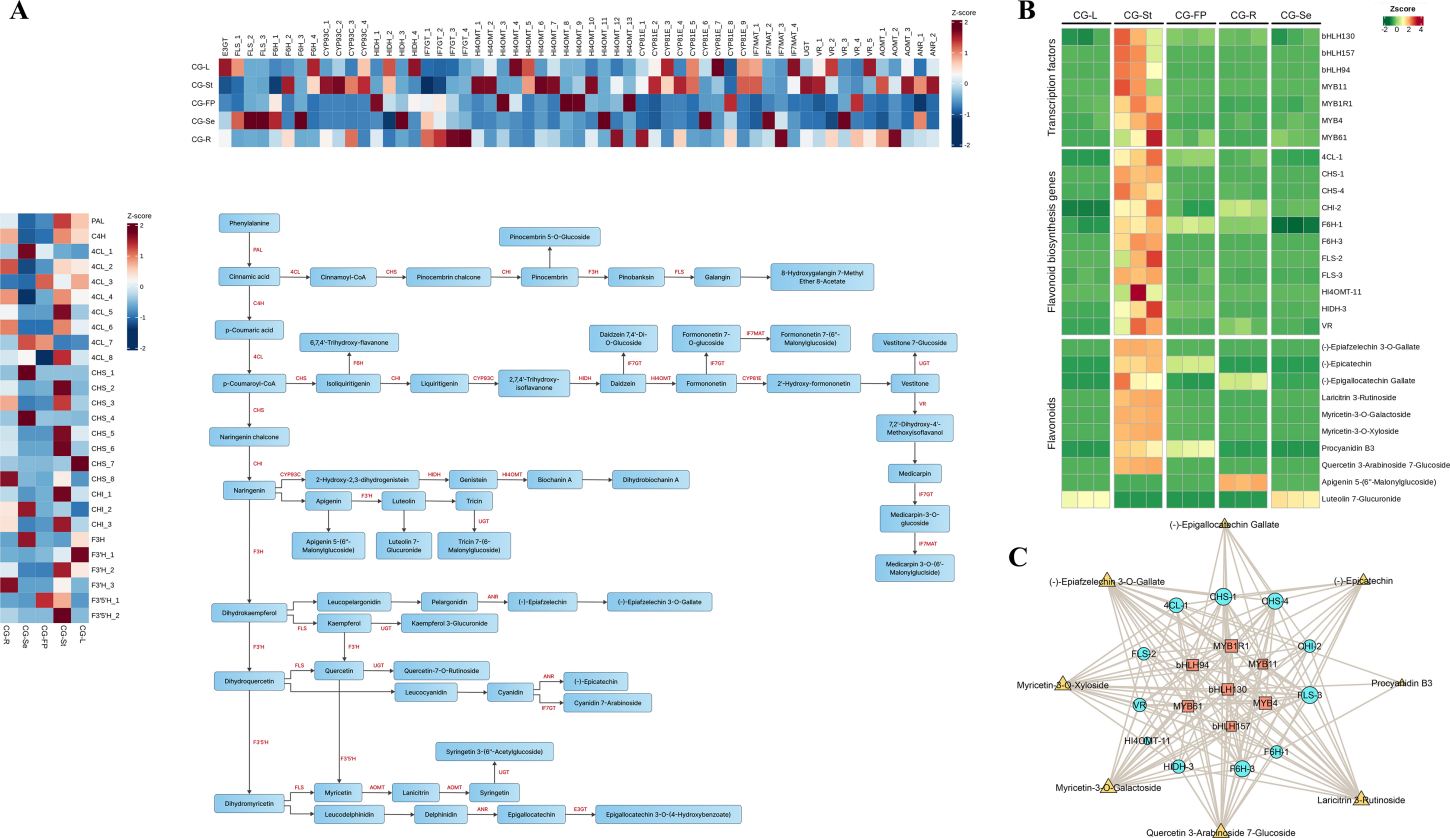
Biosynthesis and regulatory network of flavonoids. **(A)**. Metabolites and DEGs in Flavonoid biosynthesis pathway. The color gradient from blue to red indicates the expression level of DEGs from low to high. PAL, Phenylalanine ammonia-lyase; C4H, Cinnamate 4-hydroxylase; 4CL, 4-coumarate:CoA ligase; CHS, Chalcone synthase; CHI, Chalcone isomerase; F3H, Flavanone 3-hydroxylase; F3’H, Flavonoid 3’-hydroxylase; F3’5’H, Flavonoid 3’,5’-hydroxylase; F6H, Flavonol 6-hydroxylase; CYP93C, 2-hydroxyisoflavanone synthase; HIDH, 2-hydroxyisoflavanone dehydratase; HI4OMT, isoflavone 4’-O-methyltransferase; CYP81E, isoflavone 2’-hydroxylase; VR, Flavanone reductase; IF7GT, Isoflavone 7-O-glucosyltransferase; IF7MAT, Isoflavone 7-O-methyltransferase; ANR, Anthocyanidin reductase; E3GT, Flavonoid 3-O-glucosyltransferase; FLS, Flavonol synthase; AOMT, flavonoid O-methyltransferase; UGT, glycosyltransferase; **(B)**. Heatmap of the expression levels of transcription factors, structural genes and flavonoid metabolites in different tissues of *C. gladiata*; **(C)**. Correlation network diagram of transcription factors, structural genes and flavonoid metabolites (|cor| > 0.8, *p* < 0.05; transcription factors are represented by squares, structural genes by circles, and metabolites by triangles).

Among all the differential accumulated metabolites, a total of 201 metabolites were annotated as flavonoids or isoflavonoids, including 85 in Cluster 1, 52 in Cluster 2, 18 in Cluster 3, 9 in Cluster 4, 1 in Cluster 5, 20 in Cluster 6, and 16 in Cluster 7 (**Supplementary Figure 7**). Meanwhile, based on the KEGG annotation and homology alignment results of the DEGs, we identified a total of 84 genes involved in the biosynthetic pathway of flavonoid metabolites. These included 1 *PAL* gene, 1 *Cinnamate 4-hydroxylase* (*C4H*) gene, 8 *4-Coumarate: CoA ligase* (*4CL*) genes, 8 *CHS* genes, 3 *CHI* genes, 1 *Flavanone 3-hydroxylase* (*F3H*) gene, 3 *Flavonoid 3’-hydroxylase* (*F3’H*) genes, 2 *Flavonoid 3’,5’-hydroxylase* (*F3’5’H*) genes, 3 *Flavonol synthase* genes, 4 *Flavone 6-hydroxylase* genes, 2 *Anthocyanidin reductase* (*ANR*) genes, as well as other modification genes involved in flavonoid biosynthesis (**Figure 6A**). Interestingly, we found that homologous genes exhibited distinct tissue-specific expression patterns. *F3’H* catalyzes the conversion of apigenin to luteolin. The three *F3’H* genes displayed different expression patterns: *F3’H_1* (*gene-VNO77_24489*) was highly expressed mainly in leaves, followed by stems; *F3’H_2* (*gene-VNO77_24490*) was highly expressed in stems, followed by leaves and roots; whereas *F3’H_3* (*gene-VNO77_42569*) was highly expressed mainly in roots, followed by stems. The metabolites apigenin 5-(6’’-malonylglucoside) and luteolin 7-glucuronide are the derivatives of apigenin and luteolin, respectively, but showed different tissue-specific expression patterns. Apigenin 5-(6’’-malonylglucoside) was highly expressed mainly in roots, while luteolin 7-glucuronide was highly expressed mainly in stems and leaves (**Figure 6B**). ANR catalyzes the conversion of cyanidin to (-)-epicatechin. Among the two identified *ANR* genes, *ANR_1* (*gene-VNO77_05126*) showed the highest expression in seeds and stems, and *ANR_2* (*gene-VNO77_05127*) was most highly expressed in stems. The expression pattern of *ANR_1* was consistent with the relatively high accumulation of the metabolite (-)-epicatechin in seeds.

We conducted an integrative analysis of co-expression clusters, encompassing DAMs, differentially expressed structural genes, and transcription factors, to screen for key transcription factors of flavonoid biosynthetic genes. Based on the screening criteria of Pearson correlation coefficient > 0.8 and p-value < 0.05, we identified that the expression levels of 7 transcription factors and 11 structural genes were closely correlated in Cluster 3. In particular, the transcription factors *MYB4* (*gene-VNO77_16383*) and *MYB1R1* (*gene-VNO77_04235*) showed significant correlations with the structural genes *4CL_1* (*gene-VNO77_23988*), *CHS_1* (*gene-VNO77_01236*), *CHS_4* (*gene-VNO77_16715*), *CHI_2* (*gene-VNO77_11878*), *F6H_3* (*gene-VNO77_43128*), *FLS_2* (*gene-VNO77_10355*), and *FLS_3* (*gene-VNO77_31263*) (**Figure 6C**). These genes exhibited the highest expression levels in the seeds of *C. gladiata*, but relatively low expression levels in other tissues (**Figure 6B**). Cluster heatmap analysis revealed that the metabolites including myricetin-3-O-galactoside, myricetin-3-O-xyloside, quercetin 3-arabinoside 7-glucoside, and laricitrin 3-rutinoside, were more abundant in seeds and showed strong co-expression with *MYB4* (*gene-VNO77_16383*), *MYB1R1* (*gene-VNO77_04235*), *bHLH130* (*gene-VNO77_15549*), *bHLH157* (*gene-VNO77_13361*), *bHLH94* (*gene-VNO77_02851*), *MYB11* (*gene-VNO77_18081*), and *MYB61* (*gene-VNO77_18224*) (**Figure 6B**). During fruit development, the up-regulation of *4CL*, *CHS*, *CHI*, *FLS* and *F6H* gene expression could contributes to the accumulation of flavonoid metabolites. These results indicated that these transcription factors might participate in the regulation of flavonoid biosynthesis through the relevant structural genes in seeds.

### 3.9 Analysis of the regulatory network of terpenoid metabolites

**Figure 7:**
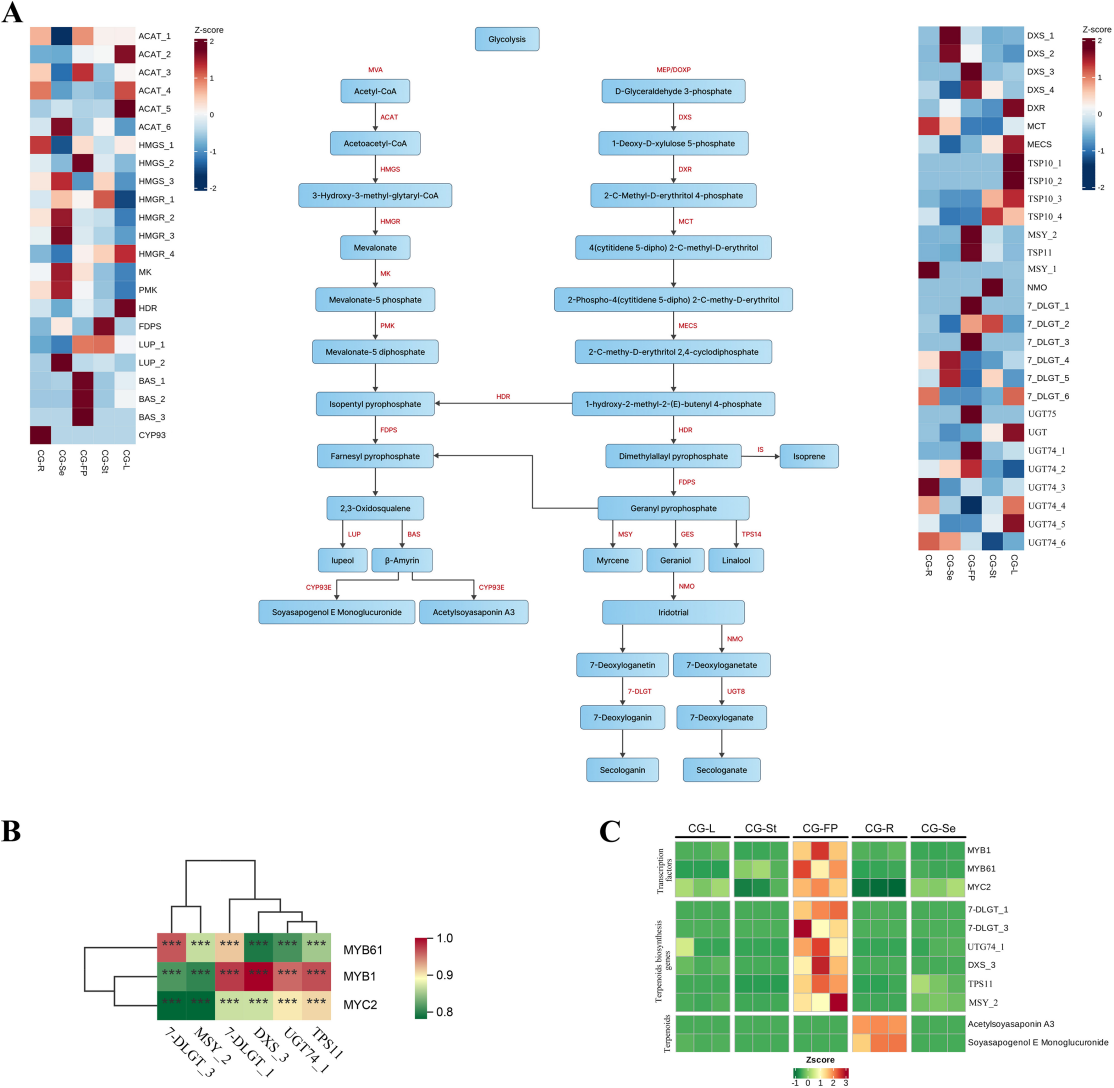
Biosynthesis and regulatory network of terpenoids. **(A)**. Metabolites and DEGs in Terpenoid biosynthesis pathway. The color gradient from blue to red indicates the expression level of DEGs from low to high. ACAT, acetyl-CoA C-acetyltransferase; BAS, beta-amyrin synthase; DXR, 1-deoxy-D-xylulose-5-phosphate reductoisomerase; DXS, 1-deoxy-D-xylulose-5-phosphate synthase; FDPS, farnesyl diphosphate synthase; GES, geranyl diphosphate diphosphatase; HDR, 4-hydroxy-3-methylbut-2-en-1-yl diphosphate reductase; HMGR, hydroxymethylglutaryl-CoA reductase; HMGS, hydroxymethylglutaryl-CoA synthase; IS, isoprene synthase; LUP, Lupeol synthase; MCT, 2-C-methyl-D-erythritol 4-phosphate cytidylyltransferase; MECS, 2-C-methyl-D-erythritol 2,4-cyclodiphosphate synthase; MK, mevalonate kinase; MSY, myrcene synthase; NMO, nepetalactol monooxygenase; PMK, phosphomevalonate kinase; TPS14, linalool synthase; UGT8, 7-deoxyloganetic acid glucosyltransferase; 7-DLGT, 7-deoxyloganetin glucosyltransferase; **(B)**. Correlation heatmap of transcription factors and structural genes; B’. Heatmap of the expression levels of transcription factors, structural genes and metabolites in different tissues of *C. gladiata*. **(C)** Heatmap of the expression levels of transcription factors, structural genes and flavonoid metabolites in different tissues of *C. gladiata*. The statistical significance of the correlations is marked on the heatmap as symbols ***(p < 0.001).

Among all the differential accumulated metabolites, a total of 147 metabolites were annotated as terpenoids, including 33 in Cluster 1, 17 in Cluster 2, 47 in Cluster 3, 17 in Cluster 4, 7 in Cluster 5, 25 in Cluster 6, and 1 in Cluster 7 (**Supplementary Figure 7**). Meanwhile, we analyzed the differential gene expression profiles of the mevalonate (MVA) pathway and the 2-C-methyl-D-erythritol 4-phosphate (MEP) pathway, which are involved in terpenoid backbone biosynthesis, to characterize the terpenoid biosynthetic pathways across different tissues. A total of 52 DEGs involved in the terpenoid biosynthetic pathway were identified (**Figure 7A**), including 6 *AACT* genes, 3 *HMGS* genes, 4 *HMGR* genes, 1 *MK* gene, 1 *PMK* gene, 4 *DXS* genes, 1 *DXR* gene, 1 *MCT* gene, 1 *MECS* gene, and 1 *HDR* gene. In the MVA pathway, 3-hydroxy-3-methylglutaryl-CoA reductase (HMGR) is the rate-limiting enzyme, whereas DXS and DXR are the key rate-limiting enzymes in the MEP pathway (Movahedi et al., 2022).The results of the cluster heatmap showed that the four HMGR genes exhibited distinct tissue-specific expression patterns. *HMGR_1* (*gene-VNO77_02361*) had the highest expression in stems, followed by seeds and fruit pericarps. *HMGR_2* (*gene-VNO77_30043*) and *HMGR_3* (*gene-VNO77_40459*) shared a similar tissue expression pattern, with the highest expression in seeds. *HMGR_4* (*gene-VNO77_44685*) showed the highest expression in leaves, followed by stems and fruit pericarps. Among the four identified differentially expressed *DXS* genes, *DXS_1* (*gene-VNO77_01613*) and *DXS_2* (*gene-VNO77_05792*) had the highest expression in seeds, whereas *DXS_3* (*gene-VNO77_24015*) and *DXS_4* (*gene-VNO77_40184*) were most highly expressed in fruit pericarps. The *DXR* gene (*gene-VNO77_24682*) had the highest expression in leaves. Two triterpenoid metabolites (acetylsoyasaponin A3 and soyasapogenol E monoglucuronide) were significantly enriched in the roots of *C. gladiata*, which was consistent with the tissue expression pattern of the *CYP93* (*gene-VNO77_17161*), suggesting that the CYP93 might be involved in regulating the synthesis of the above-mentioned metabolites.

Based on the criteria of Pearson correlation coefficient > 0.8 and p-value < 0.05, we screened the transcription factors potentially involved in regulating terpenoid biosynthesis in different clusters. In Cluster 4, we found that the transcription factor MYC2 (gene-VNO77_40240) was strongly co-expressed with the *TPS11* (*gene-VNO77_28593*), which was consistent with the findings of previous studies (Song et al., 2022). Further studies revealed that MYC2 was co-expressed with MYB1 (gene-VNO77_11271), a member of the MYB family of transcription factors, as well as with the structural genes *7-DLGT_1*(*gene-VNO77_05256*), *UGT74_1* (*gene-VNO77_35292*), *DXS_3* (*gene-VNO77_24015*), and *TPS11* (*gene-VNO77_28593*) (**Figure 7B**). In addition, the transcription factor *MYB61* (*gene-VNO77_18188*) was co-expressed with the structural genes *7-DLGT_1* (*gene-VNO77_05256*) and *7-DLGT_3* (*gene-VNO77_22234*). Compared with other tissues, these genes showed the highest expression in the fruit pericarps of *C. gladiata* (**Figure 7C**). These results indicated that the transcription factors might be involved in the regulation of terpenoid biosynthetic genes.

### 3.10 Quantitative real-time PCR validation

**Figure 8:**
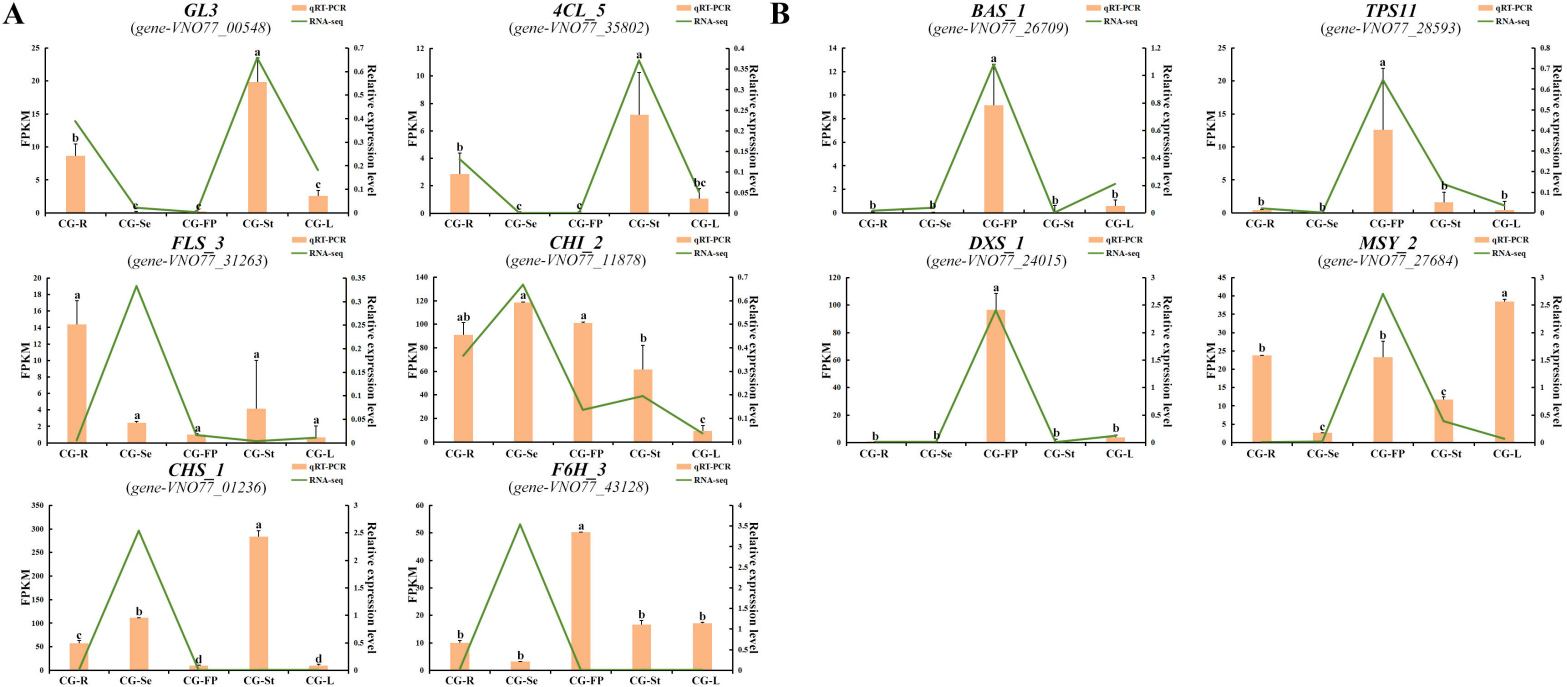
Validation of genes involved in flavonoid and terpenoid biosynthesis by qRT-PCR. **(A)** Comparison of expression trends between qRT-PCR and RNA-seq for six genes involved in flavonoid biosynthesis; **(B)** Comparison of expression trends between qRT-PCR and RNA-seq for four genes involved in terpenoid biosynthesis. Orange bars represent the relative gene expression levels detected by qRT-PCR, and green lines represent the gene expression levels from RNA-seq. The qPCR data are presented as mean ± standard error, and different lowercase letters indicate significant differences among groups (*P* < 0.05).

To further verify the RNA-seq results, 10 candidate genes involved in the biosynthesis of flavonoid and terpenoid metabolites were selected for qRT-PCR analysis. The results showed that the expression trends of different genes in the five tissues of *C. gladiata* were consistent with those in the RNA-seq data, confirming the accuracy and reliability of the transcriptome results (**Figure 8**).

## 4 Discussion

*Canavalia gladiata* is a traditional leguminous crop that has garnered widespread attention for its high application value in the food, pharmaceutical, and health product industries. Previous studies on this species have primarily focused on its pharmacological activities and the functional characterization of a small subset of metabolites (Qian et al., 2025). However, the spatiotemporal distribution patterns of flavonoid and terpenoid metabolites across different *C. gladiata* tissues remain poorly characterized, and there is a critical lack of research dissecting the biosynthetic and regulatory networks governing these metabolites from an integrated multi-omics perspective. Accordingly, in this study, we integrated metabolomic and transcriptomic datasets to reveal, for the first time, the tissue-specific accumulation patterns and transcriptional regulatory networks of flavonoids and terpenoids in *C. gladiata*. Our findings provide a robust theoretical foundation for the targeted exploitation of bioactive components and metabolic engineering of this underutilized legume.

### 4.1 Medicinal potential of flavonoids and terpenoids in *C. gladiata*

In this study, we performed a systematic assessment of phenotypic, physiological and biochemical indices across five *C. gladiata* tissues: stems, leaves, roots, seeds and fruit pericarps. Our analysis revealed that the seeds possessed the strongest antioxidant activity, whereas fruit pericarps displayed the weakest. Meanwhile, the distribution patterns of total flavonoid and total phenolic acid contents were consistent with the antioxidant activity. This finding aligns with the well-documented role of flavonoids in scavenging free radicals and inhibiting oxidative stress, indicating that the seeds might be the tissue richest in antioxidant medicinal components in *C. gladiata* (Pal et al., 2022). In addition, although the fruit pericarps had relatively low antioxidant activity compared with other tissues, their high terpenoid acid content suggests that this tissue may possess unique medicinal value. Consistent with this, previous studies have shown that extracts from immature *C. gladiate* pods (ISBP) alleviated inflammation in RAW264.7 cells by modulating the production of pro-inflammatory cytokines (Hwang et al., 2020).

### 4.2 Tissue-specific accumulation of flavonoids and terpenoids in *C. gladiata*

Metabolomic profiling identified 4,405 DAMs, with 201 annotated as flavonoids and 147 as terpenoids. We observed that four flavonoid glycosides (Myricetin-3-O-Galactoside, Myricetin-3-O-Xyloside, Quercetin 3-Arabinoside 7-Glucoside, Laricitrin 3-Rutinoside) and three flavans((-)-Epigallocatechin Gallate, (-)-Epicatechin, (-)-Epiafzelechin 3-O-Gallate) were significantly enriched in seeds compared to other tissues (**Supplementary Table 2**). These metabolites have been widely reported to exert potent antioxidant, anti-inflammatory, and antitumor activities (Joshi et al., 2024; Pal et al., 2022), suggesting that they are key bioactive constituents in *C. gladiata* seeds. Previous studies have identified a variety of flavonoid compounds in *C. gladiata* seeds, including glycosylated derivatives of kaempferol and quercetin such as kaempferol 3-O-rutinoside and quercetin 3-(6-O-acetyl-β-glucoside) (More et al., 2022). Interestingly, we found that these compounds were highly enriched not only in the seeds but also in the leaves of *C. gladiata* (**Supplementary Table 2**; **Figure 1**). The high accumulation of flavonoid compounds in leaves may reflect an important adaptive mechanism of *C. gladiata* in response to the external environment. It is well established that drought and salt stress induce flavonoid accumulation in plants; these metabolites act as antioxidants to alleviate damage caused by reactive oxygen species (ROS), and also improve osmotic balance via signal regulatory pathways (Nakabayashi et al., 2014). Furthermore, studies indicate that glycosylation modification enhances the water solubility and stability of flavonoid metabolites, and facilitates their storage in vacuolars, thereby enabling rapid mobilization to exert antioxidant and defensive functions under stress (Yonekura-Sakakibara et al., 2019). Similarly, our results showed that terpenoid metabolites displayed distinct tissue-specific accumulation patterns in *C. gladiata* (**Figure 1**). We detected a greater number of terpenoid metabolites significantly enriched in seeds compared with all other tissues, predominantly diterpenoids and sesquiterpenoids. Further functional characterization of these tissue-specific terpenoid metabolites will facilitate a deeper understanding of the key bioactive constituents in *C. gladiata* and their potential roles in plant development and medicinal activity.

### 4.3 Tissue-specific expression of key genes in flavonoid and terpenoid biosynthesis

Based on the KEGG annotation and homology alignment results of DEGs, we identified 84 structural genes involved in the flavonoid biosynthetic pathway and 52 structural genes involved in the terpenoid biosynthetic pathway. Most enzymes related to flavonoid and terpenoid biosynthesis are encoded by multi-gene families rather than by single genes, consistent with previous reports in model and crop species including *Arabidopsis thaliana* and soybean (Chen et al., 2011; Yonekura-Sakakibara et al., 2019). These genes often exhibit pronounced organ-specific or cell-type-specific expression patterns and are typically up-regulated under biotic or abiotic stress (Dixon et al., 2002). Gene duplication followed by subfunctionalization has been a driving force in legume flavonoid diversification, allowing distinct paralogs to fulfill organ-specific metabolic roles. We observed that the majority of phenylpropanoid pathway genes exhibited higher expression levels in stems of *C. gladiata*, followed by roots (**Figure 6A**). Correspondingly, a substantial number of flavonoid metabolites were significantly enriched in these tissues (**Figure 2C**), supporting coordinated regulation between gene expression and metabolite distribution. Similar stem-biased activation of phenylpropanoid metabolism has been reported in other medicinal plants (Zhu et al., 2021; Zhang et al., 2025). However, total flavonoid content was higher in leaves and seeds than in stems and roots (**Figure 1**). This discrepancy likely reflects a physiological trade-off, where the high diversity of metabolic intermediates in stems and roots does not necessarily translate to high quantitative accumulation of end products. This finding also suggests the presence of sophisticated, tissue-specific partitioning of flavonoid biosynthesis and storage in *C. gladiata*. Three flavonol glycosides, myricetin-3-O-xyloside, myricetin-3-O-galactoside and laricitrin 3-O-rutinoside, showed significant positive correlations in their abundance, and all were prominently enriched in *C. gladiata* seeds (**Figure 6B**).These metabolites are synthesized via the conserved flavonol branch of the phenylpropanoid pathway and the expression profiles of flavonoid 3’,5’-hydroxylase (F3’5’H) and flavonol synthase (FLS) co-determine the accumulation of myricetin-derived metabolites (Xing et al., 2021). Integrated transcriptomic and metabolomic analysis revealed significant co-expression between *4CL-1, CHS-1, CHS-4, CHI-2, F6H-1, F6H-3, FLS-2* and *FLS-3* and these flavonol glycosides, indicating that these genes are key candidates mediating their biosynthesis.

The expression patterns of terpenoid biosynthetic genes showed no obvious global trend, but distinct tissue-specific characteristics were observed (**Figure 7A**). Genes in the mevalonate (MVA) pathway, including *HMGS*, *HMGR*, *MK*, and *PMK*, were preferentially expressed in seeds, fruit pericarps, and roots, with low transcript levels in leaves. In contrast, genes in the methylerythritol phosphate (MEP) pathway, such as *DXR* and *MECS*, were predominantly expressed in leaves. This spatial divergence underscores the tissue-specific partitioning of terpenoid metabolism, aligning with observations in other medicinal species like *Panax ginseng* and *Camellia sinensis* (Xue et al., 2019; Chen et al., 2022). Soyasaponins, an oleanane-type triterpenoid predominantly found in leguminous plants, has been reported to exhibit lipid-lowering, antioxidant, anti-inflammatory, and antitumor activities (Bianco et al., 2018). We found that soyasapogenol E monoglucuronide was mainly enriched in roots, and was significantly co-expressed with the *CYP93* gene (**Figure 7B**). *CYP93* belongs to the cytochrome P450 family, and encodes a key rate-limiting enzyme for triterpenoid saponin biosynthesis in legumes. Specifically, in soybean, CYP93 catalyzes the early steps of soyasaponin biosynthesis, and functions in concert with downstream UDP-glycosyltransferases (UGTs) and acetyltransferases (ACTs) to generate diverse saponin derivatives (Confalonieri et al., 2021; Krishnamurthy et al., 2019).

### 4.4 Transcriptional regulatory networks of flavonoid and terpenoid metabolism

Transcription factors (TFs) play pivotal roles in regulating the biosynthesis of specialized secondary metabolites in medicinal plants (Shi et al., 2024). To explore the regulatory networks underlying flavonoid and terpenoid biosynthesis across different tissues of *C. gladiata*, we constructed a multi-omics co-expression network, which grouped genes and metabolites into seven distinct co-expression clusters. We found that genes and metabolites in cluster 3 exhibited significant co-expression specifically in seeds (**Figure 5**), and multiple transcription factors were significantly correlated with the flavonoid biosynthetic genes *4CL*, *CHS*, *F6H* and *FLS* (**Figure 6B**). *MYB11*, a member of the R2R3-MYB TF family, has been well characterized to activate the expression of flavonoid biosynthetic genes including *CHS*, *CHI*, *F3H*, and *FLS*, thereby promoting flavonoid accumulation in plants (Stracke et al., 2007). Meanwhile, we found that the transcription factors *bHLH94, bHLH130*, and *bHLH157* were significantly co-expressed with *MYB11*. Studies have shown that *R2R3-MYB*, *bHLH*, and *WD40* proteins can form the *MBW* (*MYB–bHLH–WD40*) complex in plants. This complex directly binds to the promoter regions of structural genes related to flavonoid and anthocyanin biosynthesis, thereby driving the accumulation of secondary metabolites (Bulanov et al., 2025). We therefore hypothesize that these transcription factors may interact with each other in *C. gladiata* seeds to coordinately regulate flavonoid biosynthesis. In addition, we found that the transcription factors *MYB1R1* and *MYB4* were strongly co-expressed. Mao et al. reported that *MYB1R1* expression is significantly correlated with the transcript abundance of flavonoid biosynthetic pathway genes and flavonoid metabolite content (Mao et al., 2024). Ectopic overexpression of *CstMYB1R1* in saffron petals upregulated the expression of genes involved in the flavonoid and anthocyanin biosynthetic pathways (Bhat et al., 2024). In contrast, *MYB4* is generally regarded as a negative regulator of flavonoid and phenylpropanoid metabolism. In *Arabidopsis thaliana*, *AtMYB4* can directly repress the transcription of structural genes, thereby reducing the flux of phenylpropanoid metabolism and limiting flavonoid synthesis (Banerjee et al., 2024). However, in mulberry (*Morus alba*) seeds, *MaMYB4* cooperates with *bHLH3* and *TTG1* to regulate flavonoid metabolism, preventing excessive enhancement of metabolic flux through a feedback mechanism and maintaining the balance between flavonoids and anthocyanins (Li et al., 2020b). This findings suggest that *MYB4* may play a role in maintaining flavonoid homeostasis in *C. gladiata* seeds. Regarding the regulation of terpenoid biosynthesis, our results showed that *MYC2* was co-expressed with terpenoid biosynthetic genes including *DXS*, *TPS11*, and multiple *UGTs* in *C. gladiata* fruit pericarps, and functioned synergistically with the TFs *MYB1* and *MYB61* (**Figure 7B**). Studies have demonstrated that *MYC2* is a core regulatory factor in the jasmonic acid (JA) signaling pathway, which can activate genes in the MEP pathway (e.g., *DXS, DXR* and *GGPPS*) to promote terpenoid biosynthesis (Alfieri et al., 2018; Zeng et al., 2025). Moreover, *MYC2* functions synergistically with members of the R2R3-MYB family to regulate the expression of terpenoid biosynthetic genes (Yang et al., 2020). This functional synergy suggests that *MYC2* may interact with *MYB1* or *MYB61* to modulate terpenoid accumulation in *C. gladiata*. Collectively, these findings provide novel insights into the transcriptional regulation of flavonoid and terpenoid metabolites across different *C. gladiata* tissues, and identify candidate genes for future functional validation and potential metabolic engineering applications. Further studies using dual-luciferase reporter assays, yeast one-hybrid assays, or transgenic approaches will be required to confirm the regulatory roles of these candidate TFs in *C. gladiata*.

## 5 Conclusion

*C. gladiata* is a plant with both nutritional and medicinal values. Various tissues of *C. gladiata* (roots, stems, leaves, seeds, and fruit pericarps) exhibit extensive biological effects in antioxidation, anti-inflammation and immune regulation. However, the tissue-specific differences in bioactive components and their underlying molecular regulatory mechanisms remain to be elucidated. Antioxidant activity assays showed that the seeds of *C. gladiata* had the highest antioxidant activity and total phenol content, whereas the leaves had the highest contents of total flavonoids and total terpenic acids. A total of 4,405 DAMs were identified across tissues by LC-MS analysis, revealing pronounced tissue-specific distribution patterns. The flavonoid and terpenoid biosynthesis pathways were significantly enriched in the KEGG analysis, consistent with the notion that they are key regulators underlying the metabolic divergence between seeds and other tissues. RNA-Seq analysis identified 25,597 DEGs, including 1,330 transcription factors. Subsequently, integrated correlation and k-means clustering of these data with metabolite profiles enabled the construction of regulatory networks and the classification of co-expressed metabolites and genes into 7 distinct clusters. Guided by these clusters, we uncovered potential “metabolite-transcription factor-structural gene” regulatory relationships specific to flavonoid and terpenoid metabolism in seeds and fruit pericarps, respectively. This study provides comprehensive insights into the metabolome and transcriptome of different tissues of *C. gladiata*, and laying a solid foundation for the functional development and resource utilization of this plant.

## Data Availability

The original contributions presented in the study are publicly available. This data can be found here: PRJNA1449233.

## Supplementary material

Supplementary Figure 1 OPLS-DA plots of pairwise comparisons between different groups. **(A)**. OPLS-DA plot of the comparison between stems and seeds; **(B)**. OPLS-DA plot of the comparison between seeds and mature roots; **(C)**. OPLS-DA plot of the comparison between leaves and seeds; **(D)**. OPLS-DA plot of the comparison between pod pericarps and seeds. The abscissa represents the predictive principal component; the ordinate represents the orthogonal principal component. The percentage indicates the explanation rate of the component to the dataset. Each point represents a sample, and samples from the same group are marked with the same color.

Supplementary Figure 2 Permutation test plots of OPLS-DA for pairwise comparisons between different groups. **(A)**. Permutation test plot of OPLS-DA for the comparison between stems and seeds; **(B)**. Permutation test plot of OPLS-DA for the comparison between seeds and mature roots; **(C)**. Permutation test plot of OPLS-DA for the comparison between leaves and seeds; **(D)**. Permutation test plot of OPLS-DA for the comparison between pod pericarps and seeds. The abscissa represents the model values of R²Y and Q²; the ordinate represents the frequency of the model classification effect in 200 random permutation experiments. In the figure, orange represents R²Y of the random grouping model, purple represents Q² of the random grouping model, and the values indicated by black arrows are R²X, R²Y and Q² of the original model.

Supplementary Figure 3 Bar charts of differential accumulated metabolites from pairwise comparisons between different groups. **(A)**. Bar chart of differential accumulated metabolites from the comparison between stems and seeds; **(B)**. Bar chart of differential accumulated metabolites from the comparison between seeds and mature roots; **(C)**. Bar chart of differential accumulated metabolites from the comparison between leaves and seeds; **(D)**. Bar chart of differential accumulated metabolites from the comparison between pod pericarps and seeds. The abscissa represents the Log_2_FC of differential metabolites; the ordinate represents the top 20 metabolites ranked by fold change. Red indicates up-regulated metabolite content, and green indicates down-regulated metabolite content.

Supplementary Figure 4 KEGG pathway analysis of differential accumulated metabolites from pairwise comparisons between different groups. **(A)**. KEGG pathway enrichment plot of differential accumulated metabolites from the comparison between stems and seeds; **(B)**. KEGG pathway enrichment plot of differential accumulated metabolites from the comparison between seeds and mature roots; **(C)**. KEGG pathway enrichment plot of differential accumulated metabolites from the comparison between leaves and seeds; **(D)**. KEGG pathway enrichment plot of differential accumulated metabolites from the comparison between pod pericarps and seeds. The abscissa represents the Rich Factor corresponding to each pathway; the ordinate represents pathway names. The color of the points reflects the magnitude of P-value, and the size of the points represents the number of enriched differential metabolites.

Supplementary Figure 5 Boxplots of gene expression levels in *C. gladiata* samples. The abscissa represents sample names; the ordinate represents FPKM values.

Supplementary Figure 6 Statistical chart of the number of transcription factor families. The abscissa represents transcription factor families; the ordinate represents the number of identified members of the corresponding transcription factor families in *C. gladiata*.

Supplementary Figure 7 Statistical chart of the number of flavonoid, isoflavonoid and terpenoid metabolites in different clusters. The abscissa represents different clusters; the ordinate represents the number of flavonoid, isoflavonoid and terpenoid metabolites in different clusters.
