# Supplementary figures and images for: Integrative metabolomic and transcriptomic analyses reveal the regulatory mechanisms underlying the biosynthesis of flavonoid and terpenoid metabolites in different tissues of Canavalia gladiata
Source: Front Plant Sci. 2026 Apr 15;17:1792177. doi: 10.3389/fpls.2026.1792177 (PMC13125093; doi:10.3389/fpls.2026.1792177)

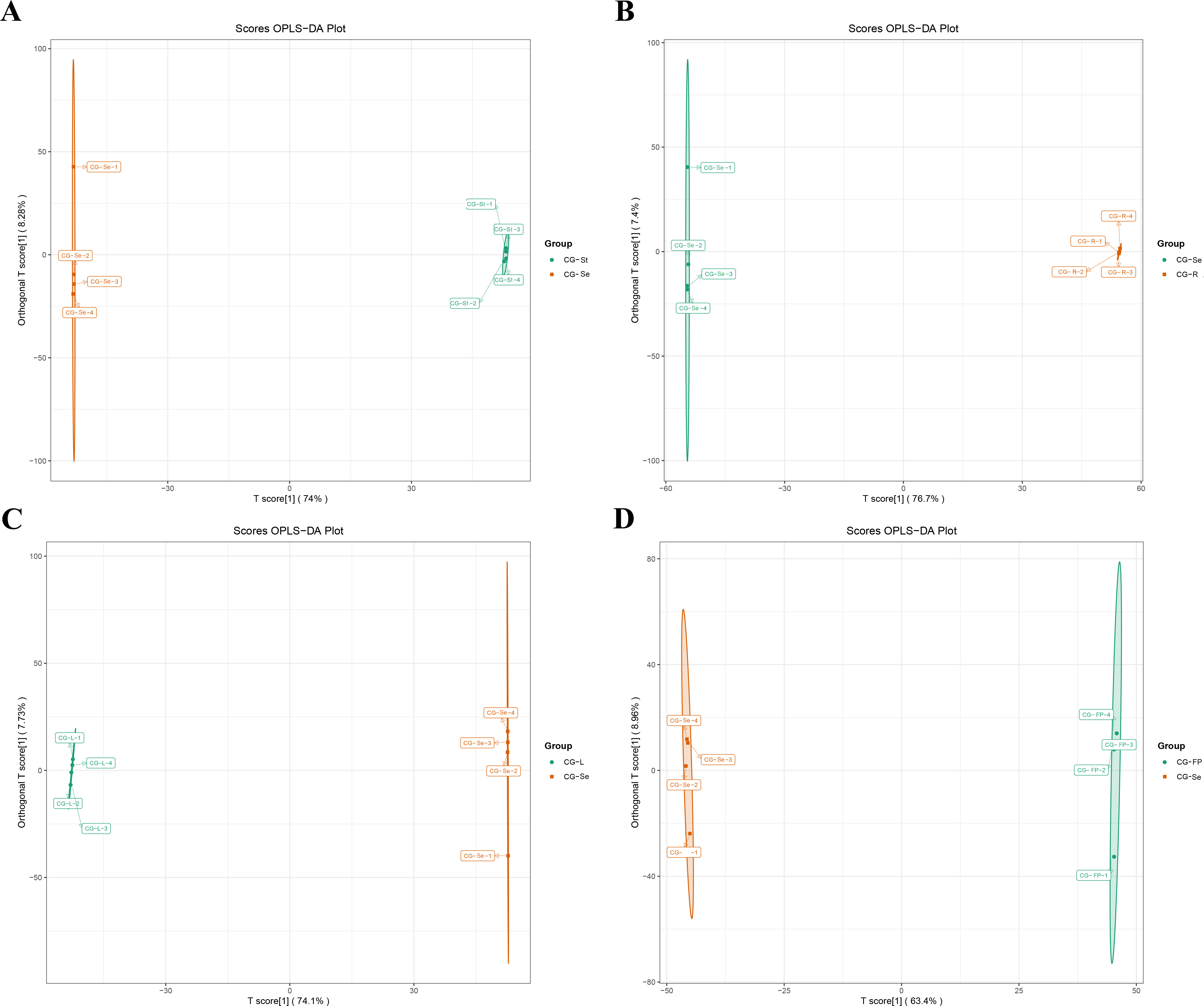

Supplement: Supplementary Figure 1 — OPLS-DA plots of pairwise comparisons between different groups. (A). OPLS-DA plot of the comparison between stems and seeds; (B). OPLS-DA plot of the comparison between seeds and mature roots; (C). OPLS-DA plot of the comparison between leaves and seeds; (D). OPLS-DA plot of the comparison between pod pericarps and seeds. The abscissa represents the predictive principal component; the ordinate represents the orthogonal principal component. The percentage indicates the explanation rate of the component to the dataset. Each point represents a sample, and samples from the same group are marked with the same color. [file Image1.jpeg]

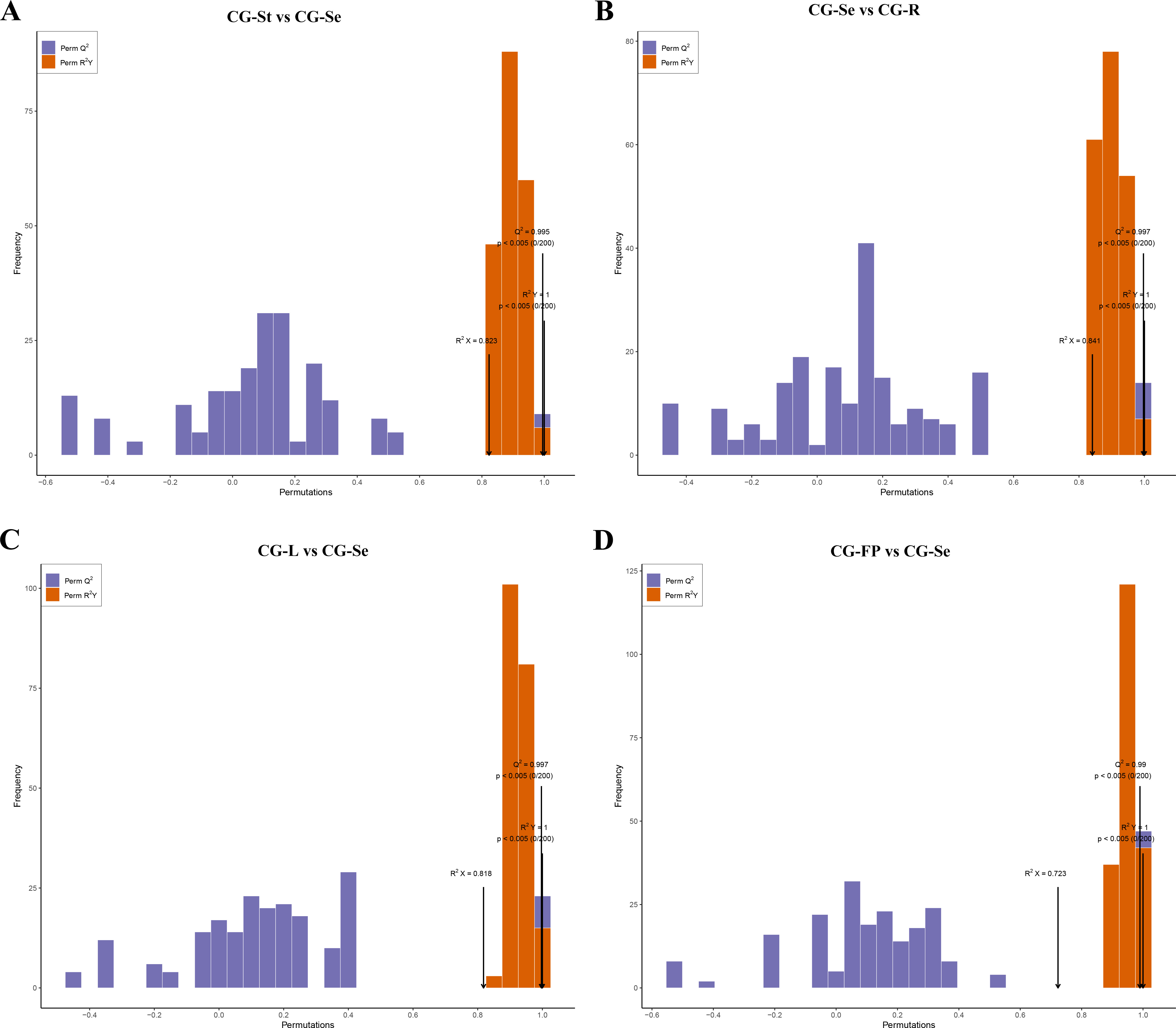

Supplement: Supplementary Figure 2 — Permutation test plots of OPLS-DA for pairwise comparisons between different groups. (A). Permutation test plot of OPLS-DA for the comparison between stems and seeds; (B). Permutation test plot of OPLS-DA for the comparison between seeds and mature roots; (C). Permutation test plot of OPLS-DA for the comparison between leaves and seeds; (D). Permutation test plot of OPLS-DA for the comparison between pod pericarps and seeds. The abscissa represents the model values of R²Y and Q²; the ordinate represents the frequency of the model classification effect in 200 random permutation experiments. In the figure, orange represents R²Y of the random grouping model, purple represents Q² of the random grouping model, and the values indicated by black arrows are R²X, R²Y and Q² of the original model. [file Image2.jpeg]

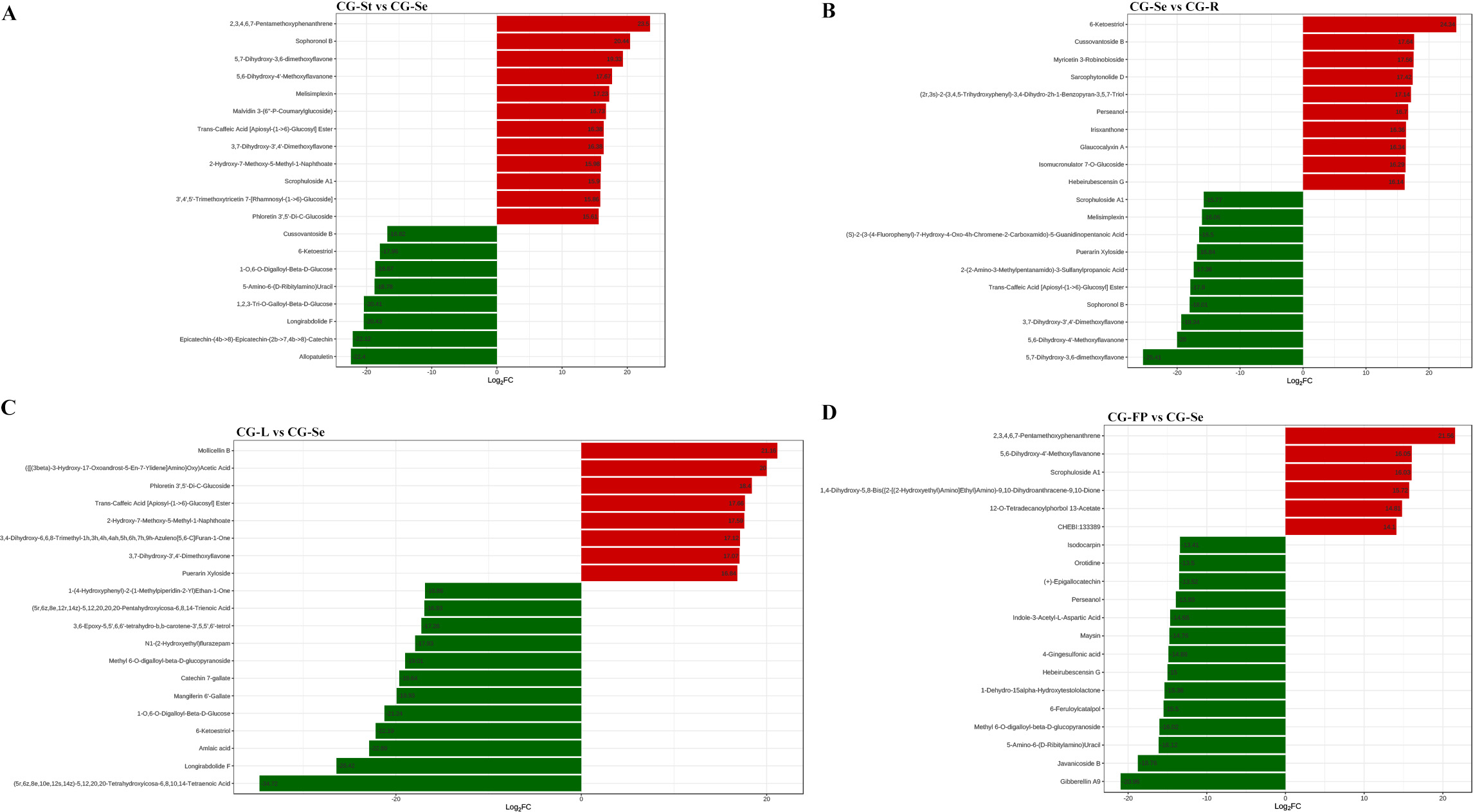

Supplement: Supplementary Figure 3 — Bar charts of differential accumulated metabolites from pairwise comparisons between different groups. (A). Bar chart of differential accumulated metabolites from the comparison between stems and seeds; (B). Bar chart of differential accumulated metabolites from the comparison between seeds and mature roots; (C). Bar chart of differential accumulated metabolites from the comparison between leaves and seeds; (D). Bar chart of differential accumulated metabolites from the comparison between pod pericarps and seeds. The abscissa represents the Log2FC of differential metabolites; the ordinate represents the top 20 metabolites ranked by fold change. Red indicates up-regulated metabolite content, and green indicates down-regulated metabolite content. [file Image3.jpeg]

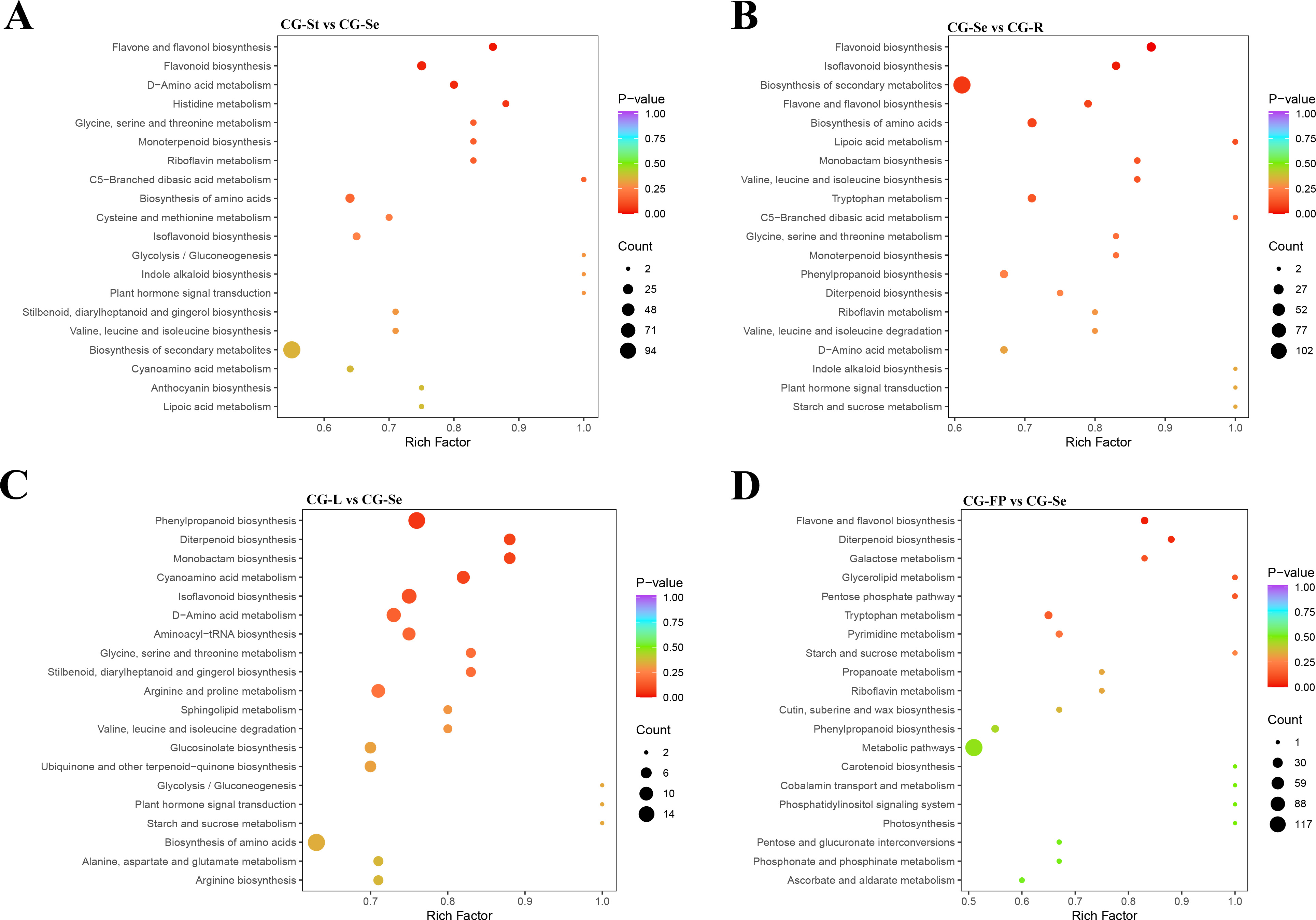

Supplement: Supplementary Figure 4 — KEGG pathway analysis of differential accumulated metabolites from pairwise comparisons between different groups. (A). KEGG pathway enrichment plot of differential accumulated metabolites from the comparison between stems and seeds; (B). KEGG pathway enrichment plot of differential accumulated metabolites from the comparison between seeds and mature roots; (C). KEGG pathway enrichment plot of differential accumulated metabolites from the comparison between leaves and seeds; (D). KEGG pathway enrichment plot of differential accumulated metabolites from the comparison between pod pericarps and seeds. The abscissa represents the Rich Factor corresponding to each pathway; the ordinate represents pathway names. The color of the points reflects the magnitude of P-value, and the size of the points represents the number of enriched differential metabolites. [file Image4.jpeg]

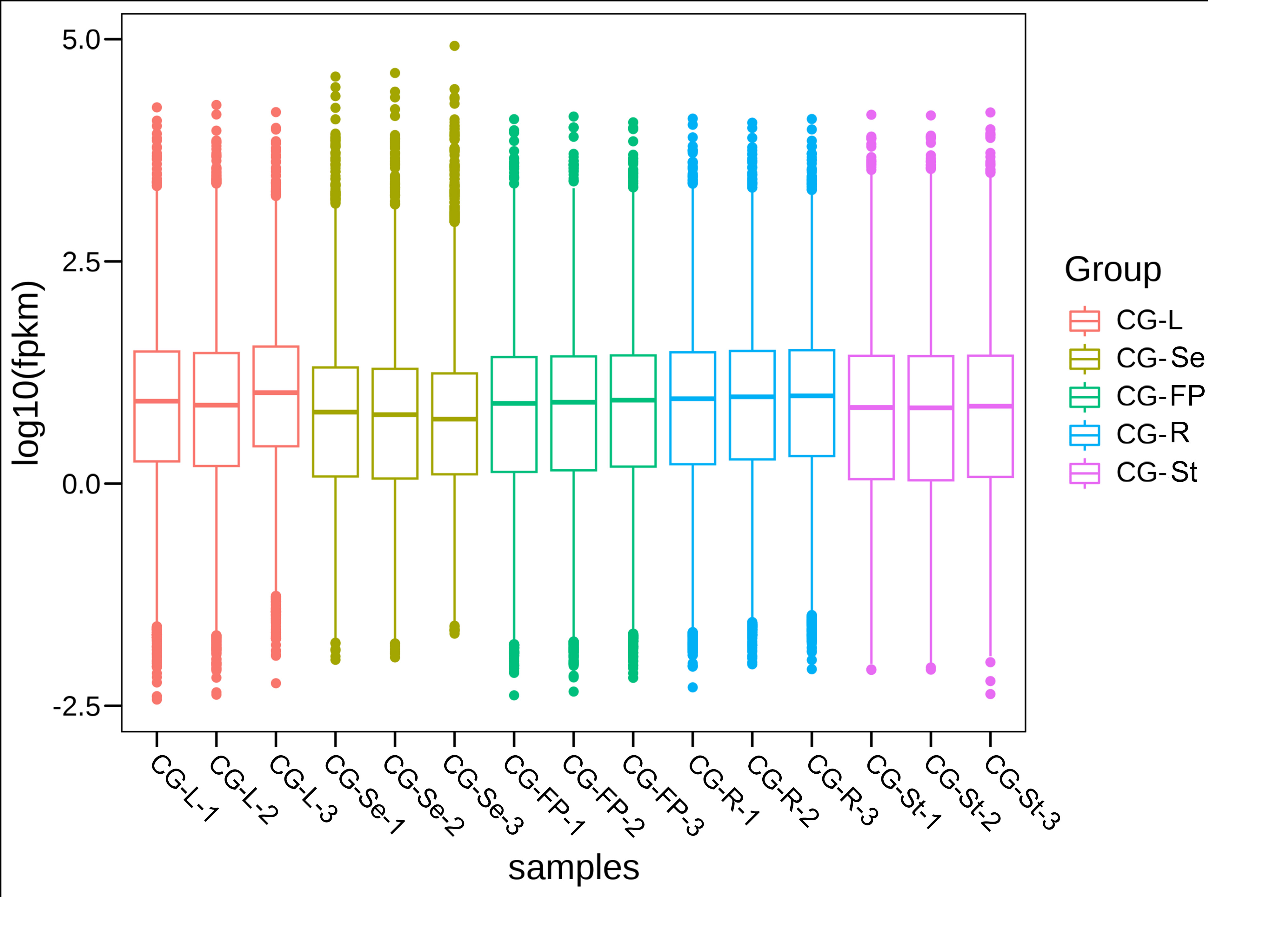

Supplement: Supplementary Figure 5 — Boxplots of gene expression levels in C. gladiata samples. The abscissa represents sample names; the ordinate represents FPKM values. [file Image5.jpeg]

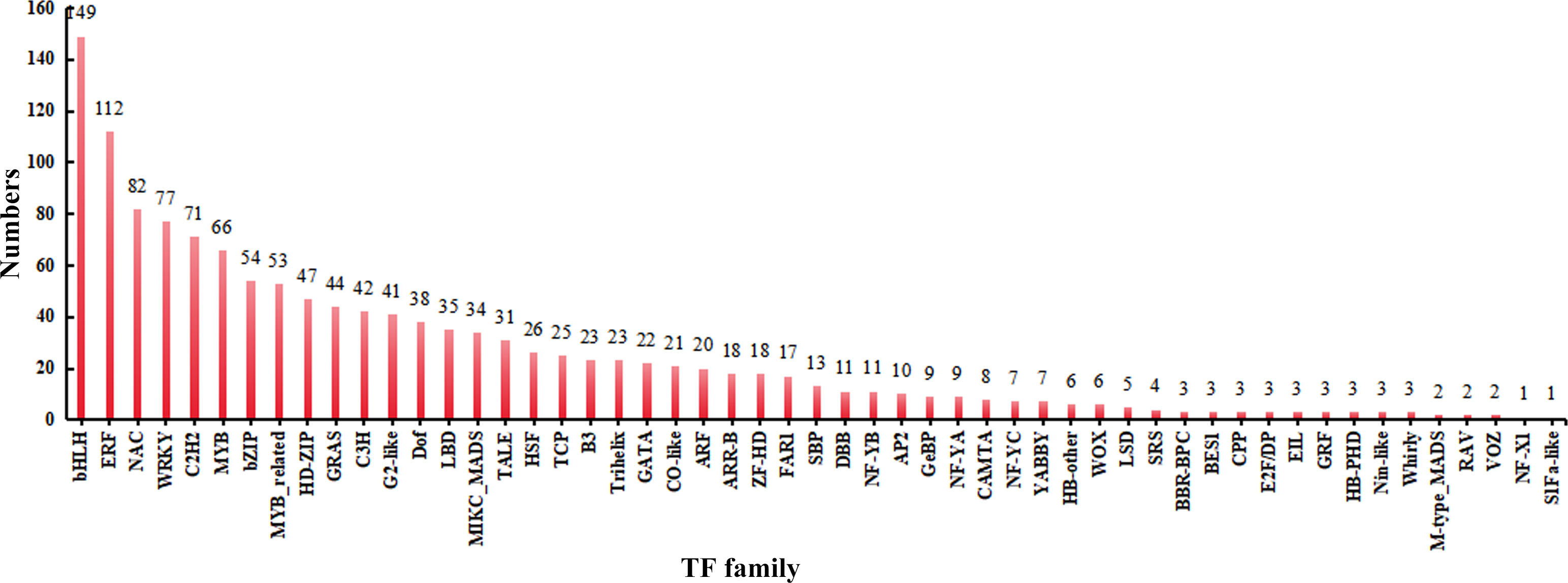

Supplement: Supplementary Figure 6 — Statistical chart of the number of transcription factor families. The abscissa represents transcription factor families; the ordinate represents the number of identified members of the corresponding transcription factor families in C. gladiata. [file Image6.jpeg]

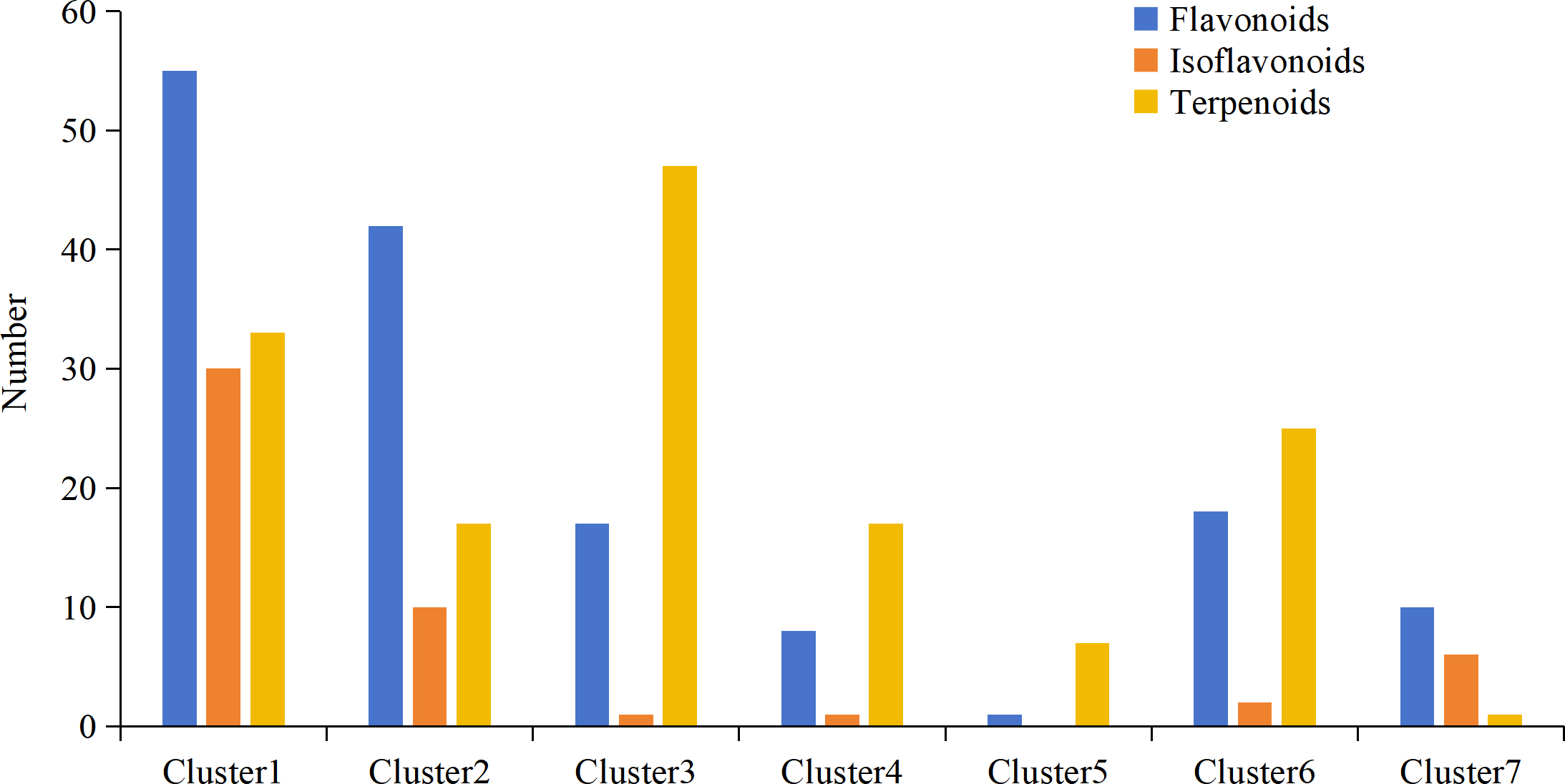

Supplement: Supplementary Figure 7 — Statistical chart of the number of flavonoid, isoflavonoid and terpenoid metabolites in different clusters. The abscissa represents different clusters; the ordinate represents the number of flavonoid, isoflavonoid and terpenoid metabolites in different clusters. [file Image7.jpeg]
